# Using circulating tumor DNA as a novel biomarker of efficacy for dose-finding designs in oncology

**DOI:** 10.1177/09622802251350457

**Published:** 2025-07-01

**Authors:** Xijin Chen, Pavel Mozgunov, Richard D Baird, Thomas Jaki

**Affiliations:** 1 MRC Biostatistics Unit, University of Cambridge, Cambridge, UK; 2 Cancer Research UK, Cambridge Centre, University of Cambridge, Cambridge, UK; 3 9147 University of Regensburg, Regensburg, Germany

**Keywords:** Bayesian adaptive trial design, dose-finding studies, biomarker, circulating tumour DNA

## Abstract

Dose-finding trials are designed to identify a safe and potentially effective drug dose and schedule during the early phase of clinical trials. Historically, Bayesian adaptive dose-escalation methods in Phase I trials in cancer have mainly focussed on toxicity endpoints rather than efficacy endpoints. This is partly because efficacy readouts are often not available soon enough for dose escalation decisions. In the last decade, ‘liquid biopsy’ technologies have been developed, which may provide a readout of treatment response much earlier than conventional endpoints. This paper develops a novel design that uses a biomarker, circulating tumour DNA (ctDNA), with toxicity and activity outcomes in dose-finding studies. We compare the proposed approach based on repeated ctDNA measurement with existing Bayesian adaptive approaches under various scenarios of dose-toxicity, dose-efficacy relationship, and trajectories of regular ctDNA values over time. Simulation results show that the proposed approach can yield significantly shorter trial duration and may improve identification of the target dose. In addition, this approach has the potential to minimise the time individual patients spend on potentially inactive trial therapies. Using two different dose-finding designs, we demonstrate that the way we incorporate biomarker information is broadly applicable across different dose-finding designs and yields notable benefit in both cases.

## 1. Introduction

### 1.1. Background

Early phase dose-finding trials, including both phase I and phase II studies are largely focussed on defining the dose and schedule of drug therapy for late-phase, pivotal trials. The traditional phase I oncology dose-finding paradigm based on toxicity without using efficacy has some undesirable consequences that the chosen dose may fail to consider the trade-off of toxicity and efficacy.^1^ Recently, there has been a significant transformation in the paradigm of drug development in oncology to integrate Phase I and Phase II trials so that drug development process may be accelerated while potentially reducing costs.^2–4^

Subsequently, the objective of the dose-finding trials is to find the optimal biologic dose (OBD) of a treatment. The concept of OBD was introduced to account for efficacy in addition to toxicity. By efficacy or activity, it normally indicates a desirable clinical outcome, which may be a composite of several events that can be scored soon enough after dose administration for adaptive decision-making to be done feasibly. For instance, this could indicate a special case of ‘activity’, such as more than 50% shrinkage of a solid tumour, engraftment of a stem cell transplant, or the resolution of an infection. By toxicity, it refers to acute events or side effects after the delivery of the study treatment.

The FDA also actively seeks ways to reform dose optimization and dose selection paradigm in oncology drug development. For instance, the Oncology Center of Excellence launched an initiative known as Project Optimus,^5^ emphasizing a core change in the dose selection that maximizes not only the efficacy of a drug but the safety and tolerability as well. Consequently, seamless phase I/II approaches allow for a more rapid and efficient transition in early phases.^6^

Seamless phase I/II designs adaptively use the (dose, efficacy, and toxicity) data from all previous patients to make decisions and select the best dose for subsequent patients. Several methods have been proposed to achieve a balance between drug safety and efficacy via a trade-off.^7–10^ One of these approaches is the EffTox design, a Bayesian adaptive dose-finding design that models correlated binary efficacy and toxicity via a joint probability function.^11^ The utility of a dose with associated posterior efficacy and toxicity probabilities measures its attractiveness and guides dose selection decisions. There are also designs where both toxicity and efficacy are independently accounted for.^12,13^

Despite the major advances in the methodology, Bayesian models for early-phase clinical trials have seen limited use in clinical practice.^14–17^ One of the practical impediments in adaptive clinical trials is that outcomes must be observed soon enough to apply decision rules to choose treatments for the next patients. In phase I, there are proposals to address the problem of delayed late-onset toxicity.^18–22^ In seamless phase I/II trial designs, there are few approaches to the problem of late-onset toxicities and efficacies. One of the existing approaches investigated a joint time to event model with delayed onset outcomes for both safety and efficacy.^13,23–27^ Data augmentation for delayed outcomes, which are regarded as missing data, is also considered.^26–28^ It seems to be more challenging in seamless phase I/II trial designs if efficacy, for instance, current clinical trials with traditional RECIST-based endpoints for efficacy, is not assessed as quickly as toxicity. The trial would then proceed at the speed determined by the efficacy outcome with the longest assessment period, and there would be an increased risk of incomplete data when one outcome is assessed and the other is waiting to be assessed. A possible solution is to use a shorter-to-evaluate activity proxy endpoint for the decision-making, where early readouts of drug response can be coupled to adaptive decision schemes. Different cases of a ‘good’ and a ‘bad’ surrogate were discussed,^29,30^ where the proposed trivariate binary model is supposed to utilize all the available data at any given time point.

In parallel, clinical trial designs of biomarker research in oncology have been popular.^31^ Mutations in cell-free DNA are highly specific markers for cancer, and this gave rise to the term ‘circulating tumour DNA (ctDNA)’,^32^ which was explored as a prognostic or predictive marker,^33,34^ and for cancer detection.^35^ In the last decade, ‘liquid biopsy’ technologies have been developed, which allow the detection of low levels of ctDNA present in plasma.^36^ In addition, there are initiatives which aim to establish international standards for ctDNA response criteria.^37^ This promotes the use of ctDNA as a promising biomarker that can obtain quantitative and qualitative comprehensive tumour DNA in a minimally invasive manner. These assays could potentially be repeated at multiple time points during the course of a patient’s treatment to provide a faster and more accurate ‘real-time’ assessment of treatment response for cancer patients on a wide range of therapies. It has been shown that in some cases – early changes in ctDNA – can predict subsequent response to treatment.^38–41^ In addition, it has been shown in several trials that early ctDNA change in response to treatment correlates with either disease-free survival or progression-free survival.^42–44^ This allows for the incorporation of novel early response or activity endpoints based on liquid biopsy-directed approaches in dose-finding studies.

In this work, we build on the EffTox approach and seek to evaluate the use of a rapidly available biomarker, ctDNA, in dose-finding studies. Specifically, we propose the Biomarker-informed EffTox (BMI-EffTox) approach, which considers additional information from continuous ctDNA measurements, and compares the BMI-EffTox and EffTox approach via a comprehensive simulation. The robustness to variability of the ctDNA values and its different trajectories are evaluated by sensitivity analyses. Wages and Tait^12^ introduced an early-phase method for trials investigating targeted agents, which falls into a broader class of extended model-based designs. To highlight the general ability of our proposal across various dose-finding designs, we extend our biomarker-informed approach to the Wages and Tait design.^12^ We do not intend to compare the EffTox design with the Wages and Tait design, as they target different objectives and follow distinct methodologies. Instead, our goal is to highlight the benefits that the proposed biomarker-informed approach can offer to both designs, which take fundamentally different approaches to modelling.

### 1.2. Motivation

**Figure 1.**
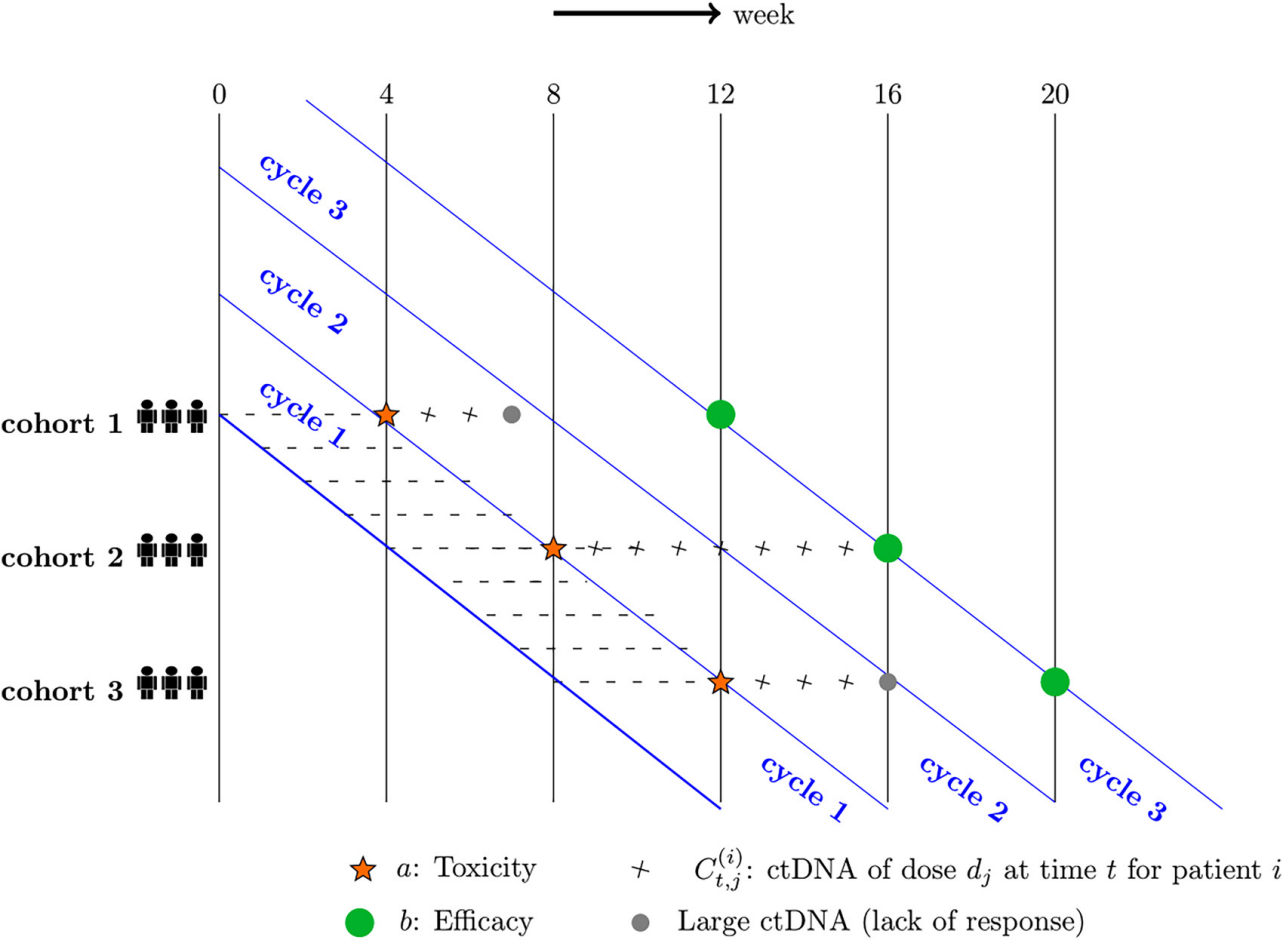
Late-onset problem in dose-finding studies.

Figure 1 illustrates the late-onset problem in an oncology trial, where there are three cohorts of patients with three patients in each. With a 28-day treatment cycle (i.e., four weeks), a cohort of patients is recruited every treatment cycle, at t=0,4,8,12⋯ . The evaluation window for each toxicity and efficacy endpoint is one cycle and three cycles, respectively. That is, the duration for assessing toxicity is one cycle (i.e., four weeks), and three cycles (i.e., 12 weeks) for assessment of efficacy outcomes given patients did not experience toxicity. A pre-specified starting dose is administered to the first cohort of patients. The next dose level will be calculated based on current data and then tested on the next recruited cohort after each cohort of patients completes the three treatment cycles (i.e., 12 weeks). The choice of toxicity and efficacy assessment window typically requires some knowledge of the timing of events. Using the first and third treatment cycle for the toxicity and efficacy window may on occasion not provide a complete representation of tolerability for targeted therapies. However, such considerations are not central to the methods we develop here.

In Figure 1, cohort 1 is firstly recruited, corresponding binary toxicity outcomes a are evaluated four weeks after recruitment at week 4 ( t=4 ), while the efficacy outcomes b are not assessable until week 12 ( t=12 ). Here, a and b are binary variables that denote if efficacy and toxicity events occurred. For a given patient, a=1 indicates the patient experienced toxicity, while a=0 implies that there is no toxicity event on the patient. Similarly, b=1 and b=0 indicate the event of efficacy and non-efficacy, respectively. Then cohort 2 and cohort 3 are enrolled four weeks and eight weeks after the recruitment of cohort 1. Corresponding toxicity outcomes are evaluated at week 8 and week 12, respectively, and efficacy outcomes are not assessable until week 16 and week 20, respectively.

One of the challenges of phase I/II trials arises from the fact that efficacy is not assessed immediately over a similar time horizon as toxicity and takes significantly longer to be evaluated. In the example in Figure 1, a patient with no toxicity event (i.e., a=0 at t=4 ) will have to wait 12 weeks to be evaluated for efficacy before resorting to some alternative treatments or sticking to the current treatment. In other words, the minimum duration of treatment is fixed at 12 weeks given non-toxicity outcomes. Activity of cohort 1 can only impact dose-decisions for cohort 4 or later in Figure 1. Besides, it might happen that a patient is losing out on receiving alternative life-saving treatment, which is of great concern in oncology.

There is more than one way to use ctDNA values before week 12, and the example in Figure 1 uses large ctDNA on an absolute scale as an indicator of ‘lack of response’. That is, if corresponding values are sufficiently large, the treatment will be deemed not useful and the patients can move to another trial/treatment before assessing efficacy endpoints. In Figure 1, ctDNA values of patients from cohort 1 and cohort 3 can be used as an indicator of ‘lack of response’ as they are large enough in this example. However, this is not the same case for patients from cohort 2. In this case, outcomes of cohort 1 can impact dose-decisions for cohort 2 and later. Thus the use of ctDNA data can help with the late-onset problem, and rapidity and efficiency of dose-finding studies can be improved.

The use of a shorter-to-evaluate activity proxy before week 12 can offer at least the following benefits: (i) The next enrolled patients can be assigned to better doses based on more information from early readouts of ctDNA; (ii) investigators can expect a faster trial; (iii) the current patients can benefit from early stopping of treatment and turn to some alternatives instead.

## 2. Methods

As our proposal of dose-finding designs builds on the EffTox approach,^11^ we begin by describing it in detail first in Section 2.1. This is then followed by the introduction of the newly proposed Biomarker-Informed EffTox approach in Section 2.2. The integration of ctDNA into the Wages and Tait design^12^ is described in Section 2.3. This approach employs relatively simple models and does not require prior specification for a large number of parameters.

### 2.1. EffTox approach

The EffTox approach is a Bayesian adaptive dose-finding design that models correlated binary efficacy and toxicity outcomes, which aims at facilitating seamless phase I/II dose-finding. It uses Bayesian models for the marginal probabilities of efficacy and toxicity at each dose and utility contours to measure the attractiveness of each dose based on the posterior probabilities of efficacy and toxicity.^45^

For J doses $y_{1},\ldots ,y_{J}$ under investigation, the transformed dose $d_{j}\in (d_{1},\ldots ,d_{J})$ is defined as $d_{j}=\text{log}\;y_{j}-\sum_{j=1}^{J}\frac{\text{log}\;y_{j}}{J}$ . The transformed dose is used as the sole explanatory variables in models for the probability of events π at dose $d_{j}$ . This transformation for the investigated doses was initially introduced when EffTox was first developed.^11^ The primary aim of this dose transformation is to maintain its independence from the specific model employed, thereby ensuring that the model evaluation remains robust regardless of the transformation method applied to the doses. Logistic regression models are used to describe the hypothesized mathematical relationships between dose and the incidence of toxicity and response, respectively. The marginal probabilities of toxicity and efficacy at dose $d_{j}$ are defined as $\text{logit}(\pi_{T}(d_{j}))=\alpha +\beta d_{j}$ and $\text{logit}(\pi_{E}(d_{j}))=\gamma +\zeta d_{j}+\eta d_{j}^{2}$ , which describe the hypothesized mathematical relationships between dose and the incidence of toxicity event and response, respectively. Denote the joint probability function as $\pi_{a,b}(\pi_{T}(d_{j}),\;\pi_{E}(d_{j}))$ , where a,b∈{0,1} indicate the occurrence of toxicity and efficacy event, correspondingly. We assume the monotonicity assumption in the toxicity probabilities $\pi_{T}(d_{j})$ with β>0 , while the parameter η for $\pi_{E}(d_{j})$ allows for a more flexible quadratic form in the dose–response relationships. To capture the correlation between binary efficacy and toxicity outcomes, an association parameter ψ is introduced in the joint probability function $\pi_{a,b}(\pi_{T}(d_{j}),\;\pi_{E}(d_{j}))$ :

(1) $$\begin{array}{rl} \pi_{a,b}(\pi_{T}(d_{j}),\;\pi_{E}(d_{j})) & =(\pi_{T}(d_{j}){)}^{a}(1-\pi_{T}(d_{j}){)}^{\left(1-a\right)}(\pi_{E}(d_{j}){)}^{b}(1-\pi_{E}(d_{j}){)}^{\left(1-b\right)}\\ & \;+(-1{)}^{\left(a+b\right)}(\pi_{T}(d_{j}))(1-\pi_{T}(d_{j}))(\pi_{E}(d_{j}))(1-\pi_{E}(d_{j}))\frac{e^{\psi }-1}{e^{\psi }+1} \end{array}$$

where (a,b) are random variables each taking values (0,1) as in Figure 1. Corresponding parameter vector in Equation (1) is θ=(α,β,γ,ζ,η,ψ) .

To select the dose with relatively higher efficacy and lower toxicity for the next cohort of patients, utility is defined as,

(2) $$u(\pi_{T}(d_{j}),\;\pi_{E}(d_{j}))=1-{\left({\left(\frac{\pi_{T}(d_{j})}{\pi_{2,T}^{*}}\right)}^{c}+{\left(\frac{1-\pi_{E}(d_{j})}{1-\pi_{1,E}^{*}}\right)}^{c}\right)}^{\frac{1}{c}}$$

where the quantity $\pi_{2,T}^{*}$ is the maximum permissible probability of toxicity when efficacy is guaranteed and the quantity $\pi_{1,E}^{*}$ is the minimum required probability of efficacy when toxicity is impossible. The value c determines the extent of the curvature of the utility contour.^11^

A likelihood-based approach could be employed, where parameters θ are estimated by applying maximum likelihood methods to the trial data. For this method to work, data heterogeneity is essential to obtain maximum likelihood estimators.^46,47^ While a likelihood-based approach is generally more demanding in terms of data configuration, particularly with an increasing number of parameters, our proposal does not add to the complexity of model fitting as it does not introduce an additional model. Instead, it offers the advantage of obtaining efficacy data earlier in the trial process.

Following the original proposal,^11^ we use a Bayesian approach to estimate the model parameters. Specifically, accumulated data about toxicity and efficacy is used to update the belief about parameters θ . Before making decisions about which dose to administer, the admissibility of all the doses in terms of both toxicity (i.e., Equation (3)) and efficacy (i.e., Equation (4)) is evaluated. That is,

(3) $$\text{Pr}\{\pi_{T}(d_{j})<{\bar{\pi }}_{T}|D\}>p_{T}$$

for toxicity, and

(4) $$\text{Pr}\{\pi_{E}(d_{j})>{\underline{\pi }}_{E}|D\}>p_{E}$$

for efficacy. The values of ${\bar{\pi }}_{T}$ and ${\underline{\pi }}_{E}$ correspond to the maximum toxicity threshold and minimum efficacy threshold for administration, and $p_{T}$ and $p_{E}$ are probability thresholds controlling exposure to overly toxic and inactive doses. Data from j th patients is $D=\{(d_{1},\;a_{1},\;b_{1},)\;\cdots \;(d_{j},\;a_{j},\;b_{j})\}$ . The investigators provide values for ${\bar{\pi }}_{T}$ , $p_{T}$ , ${\underline{\pi }}_{E}$ , and $p_{E}$ in Equation (3) and Equation (4) or these values are calibrated to yield good performance of dose-finding studies. If there is no admissible dose based on the specified admissibility criteria, the trial stops.

### 2.2. Biomarker-informed EffTox

The idea of introducing regularly available (e.g., daily, weekly or monthly) ctDNA values in dose-finding studies is achieved by regarding these early readouts as a proxy for ‘not-yet-available’ efficacy outcomes. That is, it is possible that the decisions about which dose to administer could be based on toxicity outcomes and ctDNA values when definitive efficacy outcomes are not yet observed.

(5) $$b=\left\{ \begin{array}{ll} 0, & \text{if }C_{t,j}^{\left(i\right)}\geq \epsilon \\ \text{NA}, & \text{otherwise} \end{array}\right..$$

There is no standard way to use ctDNA as a biomarker for the evaluation of treatment response in various contexts in oncology. We choose to use ctDNA as an early indicator of ‘lack of response’ in terms of efficacy as in Equation (5). This one-way information approach prevents being overly optimistic about efficacy of the administered dose and a dose cannot be deemed as ‘active’ based on ctDNA only. Consequently, higher doses are more likely to be explored, dose-escalation is more likely to be encouraged and patients are less likely to be stuck on an inactive treatment for a long time.

Regular ctDNA values are evaluated from week 4 onwards after finishing a complete treatment cycle and toxicity assessment at week 4 and week 8. In the case of ‘lack of response’ based on large ctDNA values, dose selection for the next cohort of patients can be made based on current data of toxicity and ctDNA values. While in the case of no such evidence, if ctDNA values are relatively small, the corresponding decision of dose selection will be based on only toxicity outcomes of the current patients. In this case, the corresponding marginal model shown in Equation (6) is the likelihood when there is only toxicity (See the proof in Appendix A):

(6) $$\pi_{a,b}(\pi_{T}(d_{j}),\;\pi_{E}(d_{j}))=(\pi_{T}(d_{j}){)}^{a}(1-\pi_{T}(d_{j}){)}^{\left(1-a\right)}.$$

In the example of Figure 1, seven weeks after recruitment of cohort 1, there is evidence of ‘lack of response’. Dose selection for cohort 2 patients enrolled at week 8 can be made based on toxicity a and ctDNA values $C_{t,j}^{\left(i\right)}$ of dose $d_{j}$ , which are large enough to indicate ‘lack of response’. Similarly, eight weeks after the recruitment of cohort 3 patients (at week 16), an early decision on dose selection can be made based on toxicity and ‘lack of response’. In the case of cohort 2 patients, ctDNA fails to provide evidence of ‘lack of response’ and the patients will still be on the treatment until week 16 for the evaluation of definitive efficacy results. In this way, more information from ctDNA values can be possibly used for more rapid and efficient dose-finding studies.

In contrast to the plug-in estimate for utility based on mean values of posterior efficacy and toxicity probabilities of the doses, we consider the posterior distribution of utility scores instead. That is, $\hat{u}(\pi_{E}(d_{j}),\pi_{T}(d_{j}))|D)=\frac{\int_{\Theta }u(\pi_{E}(d_{j}),\pi_{T}(d_{j}))L(\theta |D)f(\theta )d\theta }{\int_{\Theta }L(\theta |D)f(\theta )d\theta }$ , where L is the likelihood function as described in Thall and Cook,^11^ f(θ) is the prior distribution for parameters θ , and D indicates the current accumulated data. The recommended dose, namely, the OBD, is the dose that maximizes the estimated posterior mean value of utility $\hat{u}((\pi_{E}(d_{j}),\;\pi_{T}(d_{j}))|D)$ .

Overall, the BMI-EffTox with additional information from ctDNA values is linked to the EffTox approach in the way that the response outcomes are replaced by early readouts of ctDNA in the case of ‘lack of response’. In the EffTox, decision-making based on utility defined in Equation (2) is conducted at week 4 if the event of toxicity happened, otherwise current patients will have to wait for efficacy outcomes until week 12 (see Figure 1). In the BMI-EffTox, selection of the OBD can occur at week 4 and week 8 in the latter case, given there is evidence of ‘lack of response’ from ctDNA. The simple replacement of the long-term, definitive efficacy data with the ctDNA data can be an effective way considering that ctDNA has been shown to be a promising biomarker.

In summary, our proposed BMI-EffTox approach for dose-finding designs is as follows:

1. Assign the first cohort of patients to the lowest dose level $d_{1}$ .

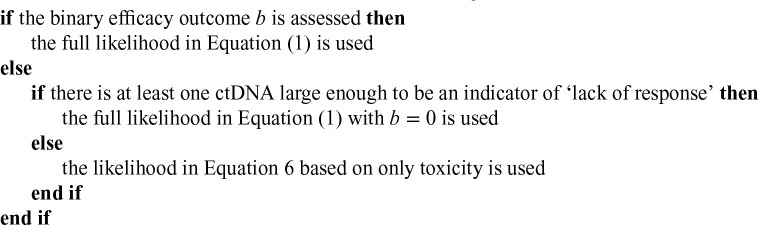

2. Update the posterior distributions of the probabilities of toxicity and efficacy at all dose levels.
3. Check the admissibility of all the doses. The trial stops if there is no admissible dose. Early stopping can occur if no dose satisfies the toxicity, efficacy, or both criteria.
4. If there is at least one dose satisfying the admissibility criteria, assign the next cohort of patients to the dose with the maximum value of utility among admissible doses. Dose skipping is not permitted.
5. Repeat from Step 2 to Step 4 until the trial is terminated early or the maximum sample size is achieved.

### 2.3. Biomarker-informed WagesTait

The original proposal by Wages and Tait was evaluated under the setting were both safety and activity data is available at the same time. As this setting does not allow for early ctDNA data to inform dosing decisions, we consider Wages and Tait’s approach when the late-onset problem (Section 1.2) is present. Henceforth, we will refer to the original proposal with the existence of the late-onset problem as WagesTait and its biomarker-informed version as biomarker-informed WagesTait (BMI-WagesTait).

In general, the WagesTait aims to assign each patient cohort to the dose estimated to be most efficacious among those with acceptable toxicity levels. This design assumes a functional dose-toxicity curve through a power model, which is common in the CRM literature. The skeleton $p_{j}$ for $d_{j}$ used is provided in Section 3.2. Similarly, the dose-efficacy relationship is modelled through a power model but includes a number of working models. Sequential Bayesian model selection is used to guide allocation decisions, and the range of working models and selection techniques enables greater flexibility in modelling the dose-efficacy relationships. The skeletons $q_{jk}$ corresponding to K=2×J−1 working models are also specified in Section 3.2. Additional model details are available in the original proposal.^12^

Our proposed BMI-WagesTait approach, developed using the same principle as for the BMI-EffTox designs, is outlined as follows:

1. Assign the first cohort of patients to the lowest dose level $d_{1}$ .
2. Identify the set of acceptable $A_{j}$ based on posterior DLT probability ${\hat{\pi }}_{T}(d_{j})$ .

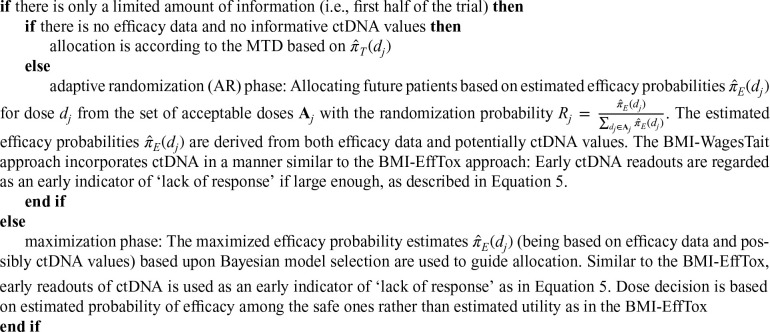

3. Check the safety based on exact binomial confidence interval ( $\pi_{T}^{-}(d_{1})$ , $\pi_{T}^{+}(d_{1})$ ) at the lowest level and futility based on exact binomial confidence interval ( $\pi_{E}^{-}(d_{j})$ , $\pi_{E}^{+}(d_{j})$ ) at the current dose level $d_{j}$ . The trial stops if there is no acceptable dose or there is no efficacious does in the maximization phase.
4. If there is at least one dose satisfying the both criteria, assign the next cohort of patients to the selected dose. Dose-skipping rule is allowed as in the original proposal.
5. Repeat from Step 2 to Step 4 until the trial is terminated early or the maximum sample size is achieved.

Instead of defining the OBD defined based on utility, as in EffTox approach described in Section 2.1, Wages and Tait^12^ employ the concept of ‘best dose’ and ‘good dose’ to evaluate the design, as introduced by Hoering et al.^48^ In this framework, the best dose is defined to maximize efficacy while assuring safety, whereas a good dose is one that meets a predefined efficacy threshold and maintains safety.

## 3. Numeric evaluation

To assess the performance of the biomarker-informed approach relative to the original designs, we conduct a comprehensive simulation study across various dose-toxicity, dose-response scenarios, and potential ctDNA trajectories.

### 3.1. Data generation

We consider a study with four doses, $d_{1}<d_{2}<d_{3}<d_{4}$ , and cycle length is assumed to be four weeks as in the motivation example in Section 1.2. Toxicity and efficacy outcomes are evaluated four weeks (i.e., one treatment cycle) and twelve weeks (i.e., three treatment cycles) after recruitment. Without additional information from early readouts of ctDNA, patients will terminate the treatment because of toxicity ( a=1 ) at week 4 or otherwise keep waiting until the assessment of efficacy at week 12.

Measurements of ctDNA are evaluated every week, while decision-making is assumed to be every four weeks as in Figure 1. In this case, the duration of treatment for patients with no toxicity outcomes ( a=0 ) can be reduced from 12 weeks to either 4 weeks or 8 weeks. Patients who terminate the treatment earlier, either because of toxicity outcomes or large ctDNA values will resort to alternative treatments or trials.

We evaluate absolute values of ctDNA for convenience in this paper, while it is also possible to evaluate the change of ctDNA values before and after the treatment on a relative scale in clinical practice. We use $C_{t,j}^{\left(i\right)}$ to notate logarithm transformed ctDNA values of patient i on dose $d_{j}$ at time point t . Weekly $C_{t,j}^{\left(i\right)}$ are considered to be normally distributed $C_{t,j}^{\left(i\right)}∼N(\mu_{t,j}^{\left(i\right)},\;\sigma^{2}),\;t\in [0,12]$ with the corresponding mean value $\mu_{t,j}^{\left(i\right)}$ and the standard deviation σ . A large value of σ indicates large between-subject variance. A quadratic relationship between time t and $\mu_{t,j}^{\left(i\right)}$ for patient i is assumed as,

(7) $$\mu_{t,j}^{\left(i\right)}=\beta_{0}+\beta_{1}\cdot t+\beta_{2}\cdot t^{2}+s^{\left(i\right)},\;\text{where}\;s^{\left(i\right)}∼N(0,\;\tau^{2}).$$

In Equation (7), τ indicates intra-subject variance in these weekly $C_{t,j}^{\left(i\right)}$ for patient i implying that patient’s responses on ctDNA over time are correlated with each other. A larger value of τ indicates smaller correlation within the same patients. For instance, patients’ responses over time are less correlated under τ=5 than under τ=0.5 .

For the relationship between ctDNA $C_{t,j}^{\left(i\right)}$ and efficacy, it is initially assumed that ctDNA can perfectly predict efficacy on the basis of the dichotomy of $C_{t,j}^{\left(i\right)}$ at a threshold ϵ . That is, high ctDNA values $C_{t,j}^{\left(i\right)}>\epsilon$ is a signal of ‘lack of response’. In this case, the value of $\pi_{E}(d_{j})$ for dose $d_{j}$ is defined as:

(8) $$\pi_{E}(d_{j})=P(\text{max}(C_{1,j}^{\left(i\right)},\ldots ,C_{12,j}^{\left(i\right)})\leq \epsilon )=\prod_{t=1}^{12}\Phi_{C_{t,j}^{\left(i\right)}}(\epsilon )$$

where Φ is the cumulative distribution function of normal distribution. Equation (8) indicates that the maximum $C_{t,j}^{\left(i\right)}$ over 12 observations has the probability of $\pi_{E}(d_{j})$ to be smaller than the threshold value ϵ . Thus, when making a decision about a dose recommendation for the next cohort of patients at t=4 , t=8 , and t=12 , the early indicator of ‘lack of response’ is defined as:

(9) $$1-\pi_{C_{\text{max}}\leq \epsilon }=1-P(\text{max}(C_{1,j}^{\left(i\right)},\ldots ,C_{t,j}^{\left(i\right)})\leq \epsilon )=1-\prod_{t=1}^{t}\Phi_{C_{t,j}^{\left(i\right)}}(\epsilon ),\;t\in (4,8,12).$$

We begin by considering this case initially to establish the maximum potential benefit of the BMI-EffTox approach. For instance, the definitive efficacy outcomes will never turn to efficacy result once early evidence from large ctDNA values indicates ‘lack of response’. The robustness of the design to the assumption of the perfect predictability of the ctDNA in Equation (8) is explored in Section 4.

### 3.2. Simulation scenarios and parameter choices

Table 1 gives a summary of all relevant parameters for different approaches, which follows the practical guidelines for EffTox in the implemented Matchpoint trial.^45^ For WagesTait, we primarily use the same parameters; for those that cannot be reasonably matched, we provide specific details below.

**Table 1.** Chosen parameters for the simulation study.

Notation	Interpretation	Value
(a) Overall parameters for the simulation study		
s	Number of simulations	1000
N	Total number of patients	45
n	Number of cohorts	15
m	Cohort size	3
J	Number of dose levels	4
(b) Design parameters for the EffTox and BMI-EffTox approach		
c	Extent of the curvature of the utility contour	2.07
σ	Between-subject variance	5
τ	Within-subject variance	0.5
ϵ	Threshold for indication of ‘lack of response’	10
$p_{E}$	Certainty required to infer dose is threshold efficable	0.03
$p_{T}$	Certainty required to infer dose is threshold tolerable	0.05
${\underline{\pi }}_{E}$	Minimum efficacy threshold	0.3
${\bar{\pi }}_{T}$	Maximum toxicity threshold	0.3
$\pi_{1,E}^{*}$	Required efficacy probability if toxicity is impossible	0.4
$\pi_{2,T}^{*}$	Required toxicity probability if efficacy is guaranteed	0.7
(c) Design parameters for the WagesTait and BMI-WagesTait approach		
K	Number of working models	2×J−1
$p_{j}$	Skeleton used for dose-toxicity relationship	(0.01, 0.08, 0.15, 0.22)
$q_{jk}$	Skeletons used for K dose-efficacy relationships	(0.10, 0.20, 0.30, 0.40)
		(0.20, 0.30, 0.40, 0.50)
		(0.30, 0.40, 0.50, 0.60)
		(0.40, 0.50, 0.60, 0.70)
		(0.20, 0.30, 0.40, 0.20)
		(0.30, 0.40, 0.50, 0.20)
		(0.40, 0.50, 0.60, 0.30)
$n_{AR}$	Size of adaptive randomization (AR) phase	22
$\psi_{E}$	Efficacy lower limit	0.3
$\psi_{T}$	Toxicity upper limit	0.33
ω(k)	No prior information for model choice	Discrete uniform prior U(K)

BMI-WagesTait: biomarker-informed WagesTait; BMI-EffTox: biomarker-informed EffTox.

For the Efftox and BMI-EffTox approaches, the prior joint distributions for the parameter vectors of θ must be specified. Ideally, the prior belief about doses is elicited by investigators or provided by clinicians. In this paper, we assume a modestly informative prior, that is, the information about dose profiles is strong enough to guide early dosing decisions but weak enough to be overridden by patient outcomes when diverging from prior beliefs.^45^ After evaluation of the operating characteristics through simulation studies, the prior is calibrated. To obtain a modestly informative prior for utility, the calibrated priors are assumed to independently follow the normal distribution. Parameters α and β for the marginal probability of toxicity are assumed to have the same standard deviation 3. Mean values are assumed to be constant −5 and 3, respectively, corresponding to the monotonicity assumption in the dose-toxicity relationship. Parameters γ , ζ , and η are assumed to follow N(0,2) , N(2,2) , and N(0,0.2) . Besides, the prior for the association parameter ψ is assumed to follow N(0,1) . The calibration for the prior commenced from what was used in the Matchpoint trial.^45^ In this trial, prior beliefs from investigators and a desired effective sample size (ESS) are combined and converted into univariate normal priors. This presents challenges for implementing this approach in practice, whereas there are only two parameters in the WagesTait approach requiring specification of priors, making it less complex in this regard.

For the WagesTait and BMI-WagesTait approaches, a normal prior with mean 0 and variance 1.34 is utilized for models for toxicity and efficacy, which can be traced back to some early work.^49^ Besides, skeletons for both toxicity and efficacy need to be specified. For working models about efficacy, a prior ω(k) for model selection among K=2×J−1 working models is needed. We proceed with a discrete uniform prior for ω(k) accounting for equal plausibility of each model, assuming there is no prior information for model choice.

For the Efftox and BMI-EffTox approaches, the maximum toxicity threshold is set to ${\bar{\pi }}_{T}=0.3$ and the minimum efficacy threshold is set to ${\underline{\pi }}_{E}=0.3$ , respectively. Assuming there are four investigated doses in the simulation study, we consider five scenarios for toxicity ( $T_{0}$ , $T_{1}$ , $T_{2}$ , $T_{3}$ , $T_{4}$ ), see Table 2a. Scenario $T_{0}$ indicates a scenario with no safe dose and so on and so forth for scenario $T_{1}$ , $T_{2}$ , $T_{3}$ , and $T_{4}$ . Similarly, five scenarios for efficacy ( $E_{0}$ , $E_{1}$ , $E_{2}$ , $E_{3}$ , $E_{4}$ ) are specified in Table 2b, corresponding to 0, 1, 2, 3, and 4 active doses on the basis of ${\underline{\pi }}_{E}$ . Additionally Table 2c provides efficacy scenarios ( $E_{5}$ - $E_{9}$ ) with an umbrella-shape. For the WagesTait and BMI-WagesTait approaches, stopping rules as in Section 2.3 are applied for safety and futility, and corresponding parameters are specified as below.

**Table 2.** Scenarios investigated in terms of dose profiles of toxicity $\pi_{T}(d_{j})$ and efficacy $\pi_{E}(d_{j})$ from $d_{1}$ to $d_{4}$ under each scenario. safe and active doses are highlighted in bold.

(a) Toxicity scenarios	$\pi_{T}(d_{j})$
$T_{0}$	(0.60, 0.65, 0.70, 0.80)
$T_{1}$	(**0.25**, 0.40, 0.50, 0.60)
$T_{2}$	(**0.10, 0.20**, 0.45, 0.55)
$T_{3}$	(**0.10, 0.15, 0.20**, 0.50)
$T_{4}$	(**0.10, 0.15, 0.20, 0.25**)
(b) Monotonic efficacy scenarios	$\pi_{E}(d_{j})$
$E_{0}$	(0.10, 0.15, 0.20, 0.25)
$E_{1}$	(0.10, 0.20, 0.25, **0.80**)
$E_{2}$	(0.10, 0.20, **0.70, 0.80**)
$E_{3}$	(0.10, **0.60, 0.70, 0.80**)
$E_{4}$	(**0.40, 0.45, 0.50, 0.60**)
(c) Umbrella-shaped efficacy scenarios	$\pi_{E}(d_{j})$
$E_{5}$	(0.05, 0.08, 0.20, 0.10)
$E_{6}$	(0.10, 0.20, **0.30**, 0.20)
$E_{7}$	(0.10, 0.20, **0.40**, **0.30**)
$E_{8}$	(0.10, **0.60**, **0.50**, **0.40**)
$E_{9}$	(**0.40**, **0.45**, **0.50**, **0.30**)

Considering both toxicity and efficacy, a specific scenario is denoted as $T_{x}E_{y}$ , where x indicates the number of safe doses under this scenario and y indicates the number of active doses. For instance, scenario $T_{3}E_{1}$ indicates a scenario where $d_{4}$ is the only toxic and active dose among all four investigated doses considering the monotonicity assumption in both toxicity and efficacy.

**Figure 2.**
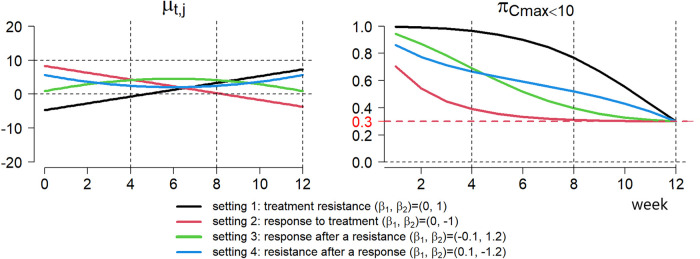
Trajectory of $\mu_{t,j}^{\left(i\right)}$ and $\pi_{\text{Cmax}<10}$ for a specific dose with $\pi_{E}(d_{j})=0.3$ under different settings and a fixed σ=5 . The value of τ=0 is fixed to obtain fixed $\mu_{t,j}$ for a particular patient.

For data generation of $C_{t,j}^{\left(i\right)}$ , the quadratic function of mean values $\mu_{t,j}^{\left(i\right)}$ in Equation (7) allows for different trajectories, which are determined by corresponding parameters $\beta_{1}$ and $\beta_{2}$ . The intercepts $\beta_{0}$ are determined such that the perfect predictability for $\pi_{E}$ (Equation (8)) is ensured. For the potential role of ctDNA as a biomarker for monitoring of response to treatment, there are different patterns at individual patient levels in clinical practice. Four settings of possible trajectories of $\mu_{t,j}^{\left(i\right)}$ are demonstrated on the left in Figure 2. For illustration, between-subject variance is fixed at τ=0 and we choose a specific dose with $\pi_{E}(d_{j})=0.3$ . The cutoff or threshold of $\mu_{t,j}^{\left(i\right)}$ for ‘lack of response’ will depend on the actual biomarker and assay used, but we will use ϵ=10 as the threshold for illustration. For the four settings evaluated, setting 1 indicates the unexpected case of treatment resistance and $\mu_{t,j}^{\left(i\right)}$ keeps increasing with time. In contrast, setting 2 is a desirable case that patients respond to treatment after drug administration and ctDNA values keep decreasing on average. Besides, it is also possible to observe a combination of these two cases where patients respond to treatment after or before a treatment resistance (setting 3 and setting 4).

Corresponding values of $\pi_{C_{\text{max}}\leq \epsilon }$ defined in Equation (9) against time t of a specific dose $d_{j}$ are illustrated on the right in Figure 2. For all investigated settings, it is less and less likely to observe the maximum value of $C_{t,j}^{\left(i\right)}$ smaller than 10 with time since dose administration. The probability $\pi_{C_{\text{max}}\leq \epsilon }$ keeps decreasing with time and ends up with $\pi_{E}(d_{j})$ at week 12 as a result of the perfect predictability shown in Equation (8).

The contribution of ctDNA to dose-finding studies is also intuitively reflected by the changing pattern of $\pi_{C_{\text{max}}\leq \epsilon }$ . The fact that $\pi_{C_{\text{max}}\leq \epsilon }\geq \pi_{E}(d_{j})$ always holds before week 12, which indicates that early readouts of ctDNA are less likely to lead to ‘lack of responses’ results than definitive binary outcomes leading to ‘non-efficacy’ results at week 12. This ensures that ctDNA is used in a conservative way. For different settings, smaller values of $\pi_{C_{\text{max}}\leq \epsilon }$ , corresponding to larger values of $C_{\text{max}}$ , contribute to dose-finding studies more than larger values of $\pi_{C_{\text{max}}\leq \epsilon }$ do. In Figure 2, ctDNA provides more information in the case of response to treatment (setting 2) than in the case of treatment resistance (setting 1). In other words, $\pi_{C_{\text{max}}\leq \epsilon }$ changes more quickly with more information provided by ctDNA. At week 4, ctDNA provides almost all of the information about $\pi_{E}(d_{j})$ in the case of response to treatment (setting 2).

### 3.3. Metrics for evaluation

To explore if decision-making for dose escalation could be improved by additional information based on ctDNA values, we focus on different metrics of the simulation results: (i) Proportion of recommendation per dose reflects the distribution of dose recommendations at the end of a trial. It also reflects how dose recommendations shift from one to another; (ii) Proportion of correct selection (PCS); (iii) Proportion of early stopping evaluates the probability that a trial ends up with an early stopping when the specified constraints defined in Equation (3) and Equation (4) are not satisfied; (iv) Duration of the treatment reflects the average number of weeks that a patient is on a given dose (see Section 1.2); (v) Proportion of toxicity reflects the possible harm on patients.

Among these metrics, the PCS and the proportion of early stopping correspond to the proportion of correct decisions, depending on whether it is a scenario with an OBD. Dose-finding studies under a scenario with an OBD would expect a higher PCS; otherwise, a higher proportion of early stopping is anticipated. Simulation results of the duration of treatment can reflect the rapidity of a trial, which is expected to be facilitated if there is no OBD. The proportion of toxicity is examined to ensure that we do not lose too much in terms of the win of efficiency of dose-finding studies.

For the WagesTait and BMI-WagesTait approaches, the PCS for both best and good dose(s) are represented. In addition to the proportion of correct decisions, the proportion of toxicity is considered. The best dose and good dose(s) for each scenario are shown in Table 5 and Table 6 in Appendix C.

### 3.4. Simulation results

We investigated simulation results under all combinations of scenarios of dose-toxicity and dose-response relationship shown in Table 2, which are displayed in terms of metrics introduced in Section 3.3. We consider the average value over all settings mentioned in Figure 2 for generalization .

####  

##### Proportion of recommendation per dose and proportion of correct decision

**Figure 3.**
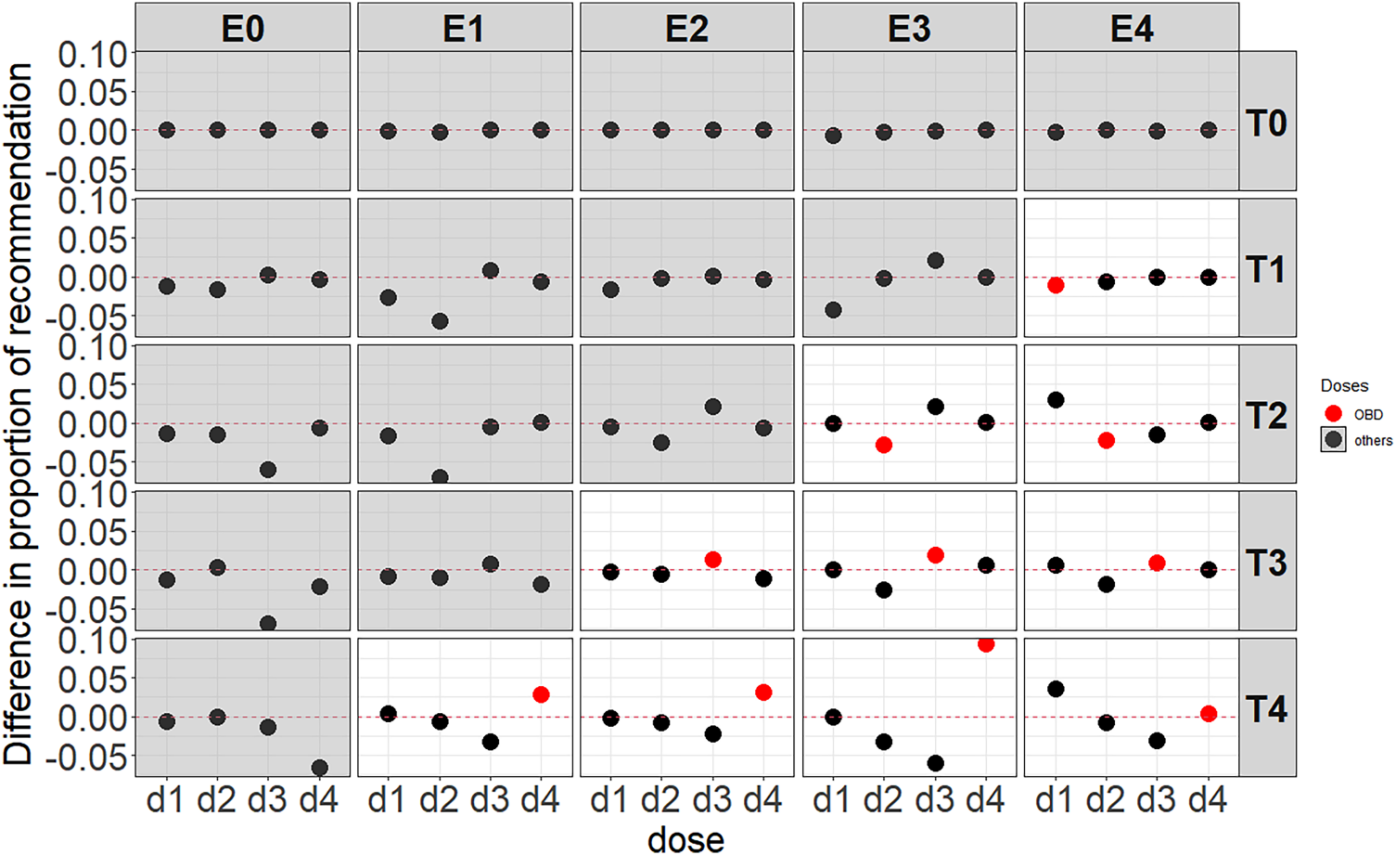
Average difference of the BMI-EffTox from the EffTox in the proportion of recommendation under monotonic efficacy scenarios with a fixed σ=5 and τ=0.5 . Scenarios with no OBD are denoted with a grey background, and the results of the difference in PCS of the OBD are denoted in red. BMI-EffTox: biomarker-informed EffTox; PCS: proportion of correct selection; OBD: optimal biologic dose.

Figure 3 illustrates simulation results of the average difference after introducing ctDNA in the BMI-EffTox with regard to the proportion of recommendation for scenarios under the assumption of monotonic efficacy curves as shown in Table 2b. The way to read Figure 3 is that negative values (i.e., decreases in the proportion of recommendation via the BMI-EffTox) in all doses are expected under scenarios with no OBD (i.e., grey background) as early stopping is expected in this case. While a positive value in the OBD (i.e., a larger PCS via the BMI-EffTox) is expected under scenarios with an OBD (i.e., white background). For scenarios with an OBD, recommendations are shifted from lower doses to higher doses, which are assumed to be more active. This effect is particularly observed under scenarios with $T_{4}$ , where all recommendations are shifted to $d_{4}$ , the most active dose and also the OBD. Scenario $T_{4}E_{4}$ is slightly different where $d_{1}$ is more likely to be recommended by the BMI-EffTox even though $d_{4}$ is the OBD under this scenario. This is acceptable as $d_{1}$ is deemed as an active and safe dose by the BMI-EffTox during the dose-escalation procedure, which is the truth. The effect of the recommendations shift to higher doses is less prominent in scenarios with $T_{3}$ . This can be explained by the utility values of investigated doses. That is, utility of the OBD is much higher than that of the rest doses (see Appendix B). In this case, the EffTox without the additional information from ctDNA will be able to identify the OBD in most of the cases. Thus the proportion of recommendation of $d_{3}$ (the OBD) is already large enough under the EffTox approach before introducing ctDNA. Specifically, up to 80% of the simulations recommend the OBD at the end of the trial (see the values in bold under $T_{3}E_{2}$ and $T_{3}E_{3}$ in Appendix D).

However, for scenarios with $T_{2}$ , such as scenario $T_{2}E_{3}$ and scenario $T_{2}E_{4}$ , the proportion of recommendations is shifted away from the OBD to a neighbouring dose. In the case of scenario $T_{2}E_{3}$ , $d_{3}$ is more likely to be recommended by the newly proposed BMI-EffTox than by the original EffTox with no ctDNA data, which has the second largest utility among all doses (see Appendix B). Similarly, in the case of scenario $T_{2}E_{4}$ , $d_{1}$ is more likely to be recommended by the proposed approach than by the original EffTox with no ctDNA data, which has the second largest utility among all doses (see Appendix B).

In Figure 3, as a result of the shift of recommendations among different doses after introducing ctDNA, the PCS, which is referred to as a selection of the OBD defined in Section 2.1 is improved in most cases. This is particularly obvious when doses are not constrained too much (i.e., scenario $T_{4}E_{3}$ ). For the ten scenarios with an OBD, there are two cases (scenario $T_{2}E_{3}$ and $T_{2}E_{4}$ ) with lower doses ( $d_{2}$ ) as the OBD, where the PCS is reduced by 1% more (2.8% under scenario $T_{2}E_{3}$ and 2.2% under scenario $T_{2}E_{4}$ , exact values are included in Appendix E).

For scenarios without an OBD, recommendations of some doses are prevented as a result of more early stopping. In clinical practice, stopping rules are commonly imposed, such that a trial can be stopped when any of the safety constraint (i.e., Equation (3)) or efficacy constraint (i.e., Equation (4)) cannot be satisfied. A trial is more likely to stop early under the BMI-EffTox approach where there are some early readouts of ‘lack of response’ results based on large ctDNA. This desirable property leads to more early stopping under almost all of the scenarios without an OBD as shown in Figure 3 (exact values are included in Appendix E). For instance, under scenario $T_{4}E_{0}$ , 8% more of the simulation results, on average, will stop before week 12 via the BMI-EffTox approach when compared with that of the EffTox approach. For scenarios with $T_{0}$ , where there is no safe dose, a trial has a high possibility of early stopping via the EffTox already, thus there are no distinct differences in the proportion of correct decisions between these two approaches in this case.

**Figure 4.**
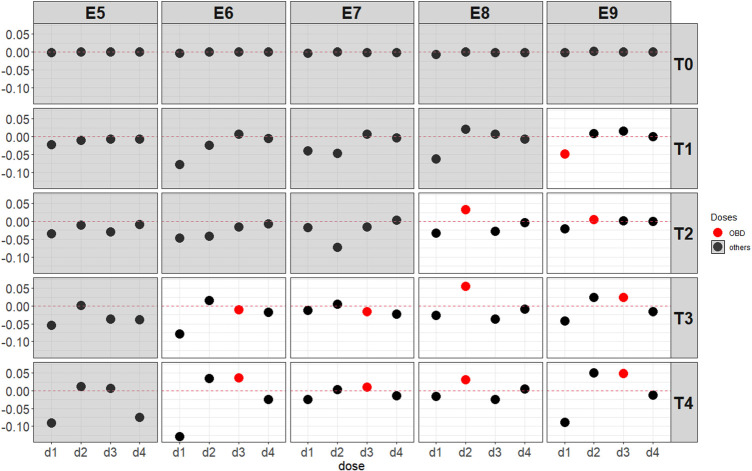
Average difference of the BMI-EffTox from the EffTox in the proportion of recommendation under umbrella-shaped efficacy scenarios with a fixed σ=5 and τ=0.5 . Scenarios with no OBD are denoted with a grey background, and the results of the difference in PCS of the OBD are denoted in red. BMI-EffTox: biomarker-informed EffTox; PCS: proportion of correct selection; OBD: optimal biologic dose.

Simulation results in Figure 4 below illustrate the scenarios under the assumption of umbrella efficacy curves as shown in Table 2c. In scenarios where an identifiable OBD exists, most cases show an increase in dose recommendations for the OBD. The PCS under scenarios with $E_{6}$ and $E_{7}$ does not increase notably, as the OBD is only marginally more active than the other doses, and the additional information does not substantially influence the outcome. In contrast, the increase in PCS under scenarios with $E_{8}$ is more pronounced due to a clear difference in activity. For the scenario $T_{1}E_{9}$ , the lowest dose, $d_{1}$ , is deemed as the OBD as it is the safest dose. The PCS is reduced by almost 5%. This corresponds to the results in Figure 3, which indicates that the BMI-EffTox leads to more early stopping when the OBD is a low dose. The impact of the magnitude of activity is discussed in Section 5 and more details about the results in Figure 4 are included in Appendix D and Appendix E.

For the EffTox approach, ctDNA encourages the dose-escalation procedures when appropriate, often leading to the recommendation of OBD by the end of the trial. This encouragement on the basis of ctDNA is more obvious under scenarios with minimal safety constraints (i.e., all doses are safe, namely, scenarios with $T_{4}$ ). There are also certain scenarios where the result is not desirable (i.e., scenario $T_{2}E_{3}$ , scenario $T_{2}E_{4}$ and scenario $T_{1}E_{9}$ ). This happens more often when the OBD is a lower dose, and the proportion of recommendations might be shifted to a neighbouring dose with a competing utility or the trial is more likely to end up with an early trial. The contribution of ctDNA might not be apparent in the case where the OBD is overwhelmingly better than the rest (i.e., scenarios with $T_{3}$ ). In these situations, the EffTox, without additional information from ctDNA, can typically identify the optimal dose without additional ctDNA input, thereby reducing the incremental benefit of ctDNA. The additional information from ctDNA will not violate the adherence to admissibility criteria or the utility-based rule for selecting the OBD, regardless of the shape of the efficacy curve.

For the WagesTait approach, Table 3 reveals similar patterns to those observed in the EffTox versus BMI-EffTox comparison (see Figure 3 and Appendix E). That is, the BMI-WagesTait improves the proportion of correct decision under most of the cases. When there is neither best dose nor good dose(s), the BMI-WagesTait approach tends to reach early stopping more frequently than the WagesTait method. For instance, the introduction of ctDNA information leads to 5% more simulations resulting in early stopping in scenarios $T_{4}E_{0}$ . In the case where there is a best dose, BMI-WagesTait generally increases the PCS by 2% up to 4.6%, with only a few cases showing a minor decrease (less than 2%) in the PCS.

**Table 3.** Average difference of the BMI-wagesTait from the wagesTait in the proportion of correct decision (**best dose**) under all scenarios with a fixed σ=5 and τ=0.5 .

	$E_{0}$	$E_{1}$	$E_{2}$	$E_{3}$	$E_{4}$
$T_{0}$	0.000 (–)	0.001 (–)	0.001 (–)	0.000 (–)	− 0.008 (–)
$T_{1}$	0.016 (–)	0.017 (–)	0.002 (–)	0.003 (–)	− 0.002 (**d1**)
$T_{2}$	0.000 (–)	0.034 (–)	0.023 (–)	− 0.001 (**d2**)	0.017 (**d2**)
$T_{3}$	− 0.034 (–)	− 0.006 (–)	0.044 (**d3**)	0.032 (**d3**)	0.033 (**d3**)
$T_{4}$	0.050 (–)	0.022 (**d4**)	− 0.016 (**d4**)	0.026 (**d4**)	0.005 (**d4**)
	$E_{5}$	$E_{6}$	$E_{7}$	$E_{8}$	$E_{9}$
$T_{0}$	0.000 (–)	0.001 (–)	0.000 (–)	− 0.001 (–)	− 0.008 (–)
$T_{1}$	0.005 (–)	0.032 (–)	0.021 (–)	0.023 (–)	0.000 (**d4**)
$T_{2}$	0.040 (–)	0.020 (–)	− 0.009 (–)	0.018 (**d2**)	0.017 (**d2**)
$T_{3}$	0.002 (–)	0.046 (**d3**)	0.012 (**d3**)	0.007 (**d2**)	0.034 (**d3**)
$T_{4}$	0.013 (–)	0.012 (**d3**)	0.028 (**d3**)	0.016 (**d2**)	0.027 (**d3**)

For scenarios without a best dose, the proportion of early stopping is illustrated, while for scenarios with a best dose, PCS is demonstrated. BMI-WagesTait: biomarker-informed WagesTait; PCS: proportion of correct selection.

Overall, the biomarker information supports both the EffTox and WagesTait models in a similar manner, contributing similarly to improved decision in both designs. Across different baseline designs, ctDNA leads to a maximum 8% increase in early stopping and a 10% improvement in the proportion of correct decisions (PCS) in the BMI-EffTox model. In the BMI-WagesTait model, ctDNA improves early stopping by up to 5% and PCS by up to 4.6%. Additional findings on the proportion of correct decision for good dose are provided in Appendix C, showing rather similar conclusions. We find that the advantage of incorporating ctDNA remains consistent in identifying both the best and good doses.

##### Duration of treatment

**Figure 5.**
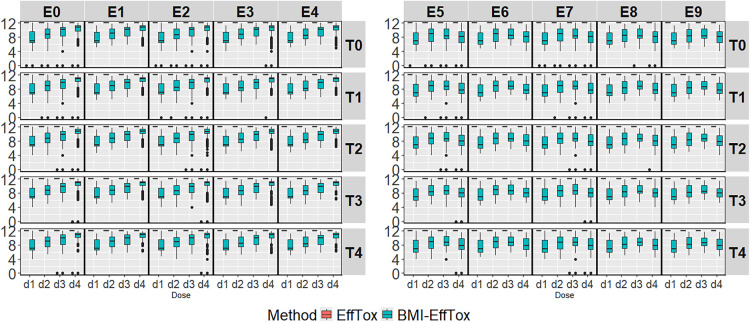
Treatment duration for the biomarker-informed EffTox (BMI-EffTox) and EffTox models across monotonic and umbrella-shaped efficacy scenarios with a fixed σ=5 and τ=0.5 .

For the rapidity of dose-finding studies, the duration of treatment is reduced after introducing ctDNA (see Figure 5), which allows more early stopping of doses showing evidence of the ‘lack of response’ discussed above.

The duration of all safe doses is fixed at 12 weeks in the EffTox approach as discussed in Section 1.2. Figure 5 illustrates the distribution of the duration of doses under all of the investigated scenarios via the EffTox and the BMI-EffTox approaches. In general, there is a reduction of the duration of treatment in the BMI-EffTox in the cases of all doses under all investigated scenarios. This is particularly observed in doses with smaller $\pi_{E}(d_{j})$ , i.e., $\pi_{E}(d_{1})=0.10$ under $S_{1}$ (see Table 2b), the duration of treatment is reduced by up to five weeks in the BMI-EffTox approach (See more details about the duration of treatment in Appendix F). This is desirable as patients can avoid struggling with a treatment that does not work at all. In addition, ctDNA helps to tell the differences between active and inactive doses, thus the reduction of the duration of treatment is significantly different in between. For instance, under scenarios with $E_{2}$ , the duration of lower doses $d_{1}$ and $d_{2}$ are reduced by no less than four weeks, while that of higher doses $d_{3}$ and $d_{4}$ are reduced by less than two weeks. In scenarios with umbrella efficacy profiles, the BMI-EffTox approach reduces the duration for all investigated doses as well. Notably, the reduction is more pronounced for inactive doses compared to active doses.

The procedure of dose-escalation might be accelerated based on the results of the ‘lack of response’, and ctDNA might encourage the exploration of higher doses. However, it might happen that an active dose leads to high values of ctDNA occasionally and is mistakenly deemed as an inactive dose. Current patients on this dose might suffer from the wrong early stopping of the treatment and lose the chance of staying on effective treatment. Meanwhile, a higher dose, which is potentially more active but more toxic, might be recommended to the next cohorts.

Simulation results of the proportion of correct decisions and the duration of treatment show the gains in rapidity and efficiency in dose-finding studies. The process of dose escalation is governed by a fundamental conflict since both the therapeutic aim and the safety concern should be considered simultaneously for cancer patients. On the one hand, there is a need to go slowly in order to avoid a sudden jump from no observable toxicity to a lethal dose. For instance, a slow dose-escalation procedure is preferred to avoid an overdose if OBD is at a lower dose. On the other hand, there is a need to go rapidly so that large numbers of patients are not treated at ineffective doses.

To explore whether the encouragement of the dose-escalation procedure by the BMI-EffTox is always tolerate, simulation results of the proportion of toxicity are evaluated. Under all of the investigated scenarios, the proportion of toxicity is similar between the EffTox and the BMI-EffTox and between the WagesTait and the BMI-WagesTait as shown in Appendix G. Large increases in proportion of toxicity are rarely observed after introducing ctDNA, suggesting that ctDNA can be safely integrated into dose-finding studies.

## 4. Imperfect predictability

Above, it was assumed that binary efficacy response and ctDNA are perfectly correlated to understand the largest benefit the biomarker-informed approach can attain over the original proposal. In this section, we relax this assumption that the prediction of ‘lack of response’ based on ctDNA might be either an underestimation or an overestimation of the real value of $\pi_{E}(d_{j})$ . We focus solely on the results of the EffTox approach under monotonic scenarios, as the simulation results for WagesTait approach above are similar anyway.

To explore the contribution of ctDNA when relaxing the assumption of perfect predictability of ctDNA to the efficacy, we generated ctDNA based on

(10) $${\tilde{\pi }}_{E}(d_{j})=P(\text{max}(C_{1,j}^{\left(i\right)},\ldots ,C_{12,j}^{\left(i\right)})\leq \epsilon )=\prod_{t=1}^{12}\Phi_{C_{t,j}^{\left(i\right)}}(\epsilon )$$

where ${\tilde{\pi }}_{E}(d_{j})=\pi_{E}(d_{j})+\delta$ and δ∈(−0.09,−0.05,0.01,0,0.05,0.1,0.2) for dose $d_{j}$ . In this case, a larger absolute value of δ indicates a worse predictability of ctDNA.

**Figure 6.**
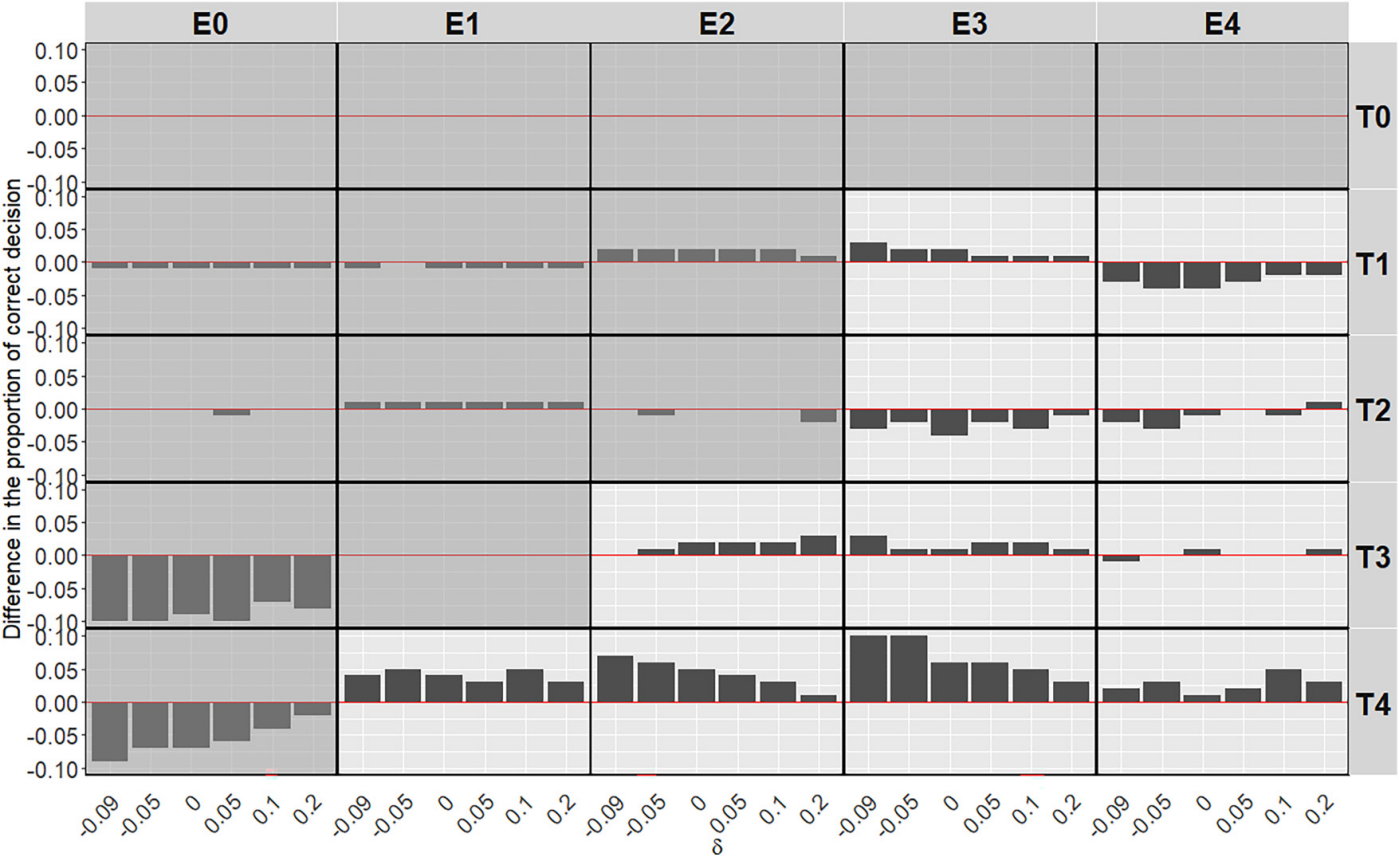
Average difference of the BMI-EffTox from the EffTox in the proportion of correct decisions under monotonic efficacy scenarios with different δ under σ=5 and τ=0.5 . Scenarios with no OBD are denoted with a grey background, and scenarios with an OBD are denoted with a white background. BMI-EffTox: biomarker-informed EffTox; OBD: optimal biologic dose.

In Figure 6, the introduction of ctDNA in the BMI-EffTox encourages early stopping under scenarios without an OBD. While in the case of scenarios with an OBD, the PCS is improved in most cases regardless of δ . This is consistent with the results in Figure 3.

For scenarios with $T_{4}$ , under which it is most likely to observe the impact of ctDNA on the proportion of recommendation per dose (as shown in Figure 3), smaller values of δ , corresponding to smaller ${\tilde{\pi }}_{E}(d_{j})$ , will lead to larger average difference in the PCS systematically. It indicates that there is more information brought by ctDNA in the case of larger underestimation. This is a result of the working mechanism of ctDNA, as a ‘one-way informative’ proxy. Specifically, ctDNA is more likely to encourage the dose-escalation procedure with more ‘lack of response’ results in the case of underestimation of the probability of definitive efficacy outcomes $\pi_{E}(d_{j})$ .

For scenarios with $T_{3}$ , average difference in the PCS between two approaches are very similar under different δ . This is because the OBD is substantially better than the rest in terms of utility (see Appendix B), ctDNA in the BMI-EffTox thus is less likely to contribute to the dose-escalation procedures. For scenarios with $T_{1}$ and $T_{2}$ , contribution of ctDNA is limited as a result of safety constraints and thus the impact of δ is not that obvious on final results of the average difference in the PCS between two approaches.

For the proportion of early stopping under scenarios without an OBD, the benefit of more frequent early stopping is particularly obvious when there is an underestimation in ${\hat{\pi }}_{E}$ . This, again, is the result of the way of using ctDNA as a ‘lack of response’ indicator.

**Figure 7.**
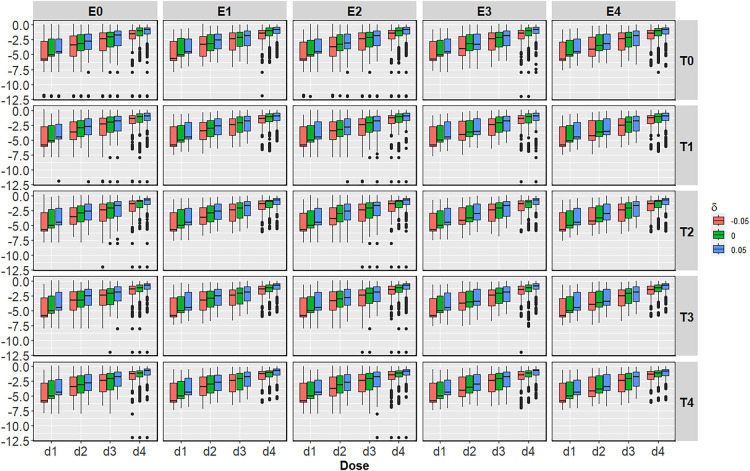
Treatment durations for the biomarker-informed EffTox (BMI-EffTox) and EffTox models under monotonic efficacy scenarios with σ=5 , τ=0.5 , and three different values of δ .

The reduction of the duration of treatment in the BMI-EffTox happens under all scenarios regardless of the value of σ . Simulation results under three values of δ ( δ=−0.5,0,0.5 ) are chosen for illustration in Figure 7. Similar to the conclusion in Figure 5, the duration of lower doses is more likely to be reduced after introducing ctDNA than that of higher doses regardless of the value of δ . Again, as an early indicator of ‘lack of response’, ctDNA tends to tell which doses are not active enough during the trial. Among different values of δ , more information brought by ctDNA under smaller δ leads to a larger reduction of the duration of the BMI-EffTox from the EffTox, and this applies to all scenarios and all doses.

## 5. Discussion

This study aimed to incorporate a novel efficacy biomarker, ctDNA, into dose-finding studies together with toxicity and efficacy data. The clinical context addresses the late-onset problem, with different observation windows for toxicity and efficacy outcomes. We compared the EffTox^11^ and WagesTait^12^ approaches with their biomarker-informed counterparts under various assumptions regarding dose-toxicity, dose-response scenarios, and possible ctDNA trajectories.

The pace of the uptake and implementation of adaptive dose-finding trial designs, such as the EffTox approach, have lagged behind advancements in the statistical literature introducing novel methodologies. Implementing the EffTox approach poses several challenges, including the specification of investigated doses, the determination of prior distributions for parameters, and the elicitation of targeted quantities from investigators and clinicians. Discussions on utility approaches and elicitation method can be found the in literature.^8,50^ Despite these practical challenges, our proposed method aims to utilize additional biomarker information to enhance the efficiency and speed of dose-finding studies. While we used the EffTox design as an example for incorporating biomarker information, similar ideas can be applied to any dose-finding approach that balances safety and efficacy. In response to reviewer feedback, we successfully integrated ctDNA into the WagesTait approach, which offers advantages in terms of model simplicity and ease of parameter specification. Simulation results demonstrate that the novelty of our approach lies in its independence from the actual model and its applicability to different existing methodologies.

We employed a Bayesian adaptive dose-finding trial design in the proposal. The likelihood-based approach would be more demanding in the sense of data configuration, particularly with an increasing number of parameters, as more data is required in the initial stage to initiate the model. For instance, two DLTs (dose-limiting toxicities) and two non-DLTs might be required in the first run-in stage.^51^ Extensive discussions on the number of parameters and model complexity exist.^52,53^

Our simulation results show that additional information from ctDNA can significantly reduce the duration of treatment and increase the efficiency of trial design. In the former case, the duration of inactive doses is reduced tremendously more than that of active doses. The latter case includes the proportion of early stopping and the PCS accordingly. Other than the benefits obtained from using ctDNA, even with the encouragement of dose-escalation procedure after introducing ctDNA in the BMI-EffTox and BMI-WagesTait, there is no obvious increase in the proportion of toxicity. It shows that the incorporation of ctDNA leads to more rapid and efficient dose-escalation schemes. The improvement in rapidity and efficiency resulting from the additional ctDNA information of applies to scenarios characterized by either monotonic or umbrella efficacy curves.

We want to point out that there are some related works for similar settings, considering the impact of the level of surrogacy in the joint model. For instance, it was emphasized that the quality of surrogate markers, which is available at any given time point, is an important factor in finding an optimal dosage.^29,30^ Our work is more specifically targeted for the promising biomarker, ctDNA, thus different assumptions, such as the perfect predictability and the evaluation of various outcomes at different time points are distinct from related works. For this reason, a direct comparison of the performance between our approach and related work might not be sensible and fair enough.

In the case that ctDNA fails to predict efficacy outcomes perfectly, results show systematic bias corresponding to ${\tilde{\pi }}_{E}(d_{j})$ . For the PCS, an underestimation of the probability of efficacy $\pi_{E}(d_{j})$ is more likely to lead to greater encouragement of dose escalation. Similarly, underestimation of real efficacy will lead to a larger reduction of the duration of treatment.

The levels of ctDNA in different clinical contexts were not yet accurately defined, it is essential that the biology of ctDNA is identified and validated in advance. For the robustness of the proposed approach, we consider sensitivity analysis in this paper: (i) Between-subject variance of ctDNA values σ (see Appendix H); (ii) intra-subject correlation of ctDNA values τ (see Appendix I). Results show desirable conclusions that different information brought by ctDNA contributes in various ways to dose-finding designs. Further work is needed in order to extend this approach using a data augmentation approach upon the availability of trinary outcomes (toxicity, efficacy, and regular biomarker) at different time points.

## Supplemental Material

sj-pdf-1-smm-10.1177_09622802251350457 - Supplemental material for Using circulating tumor DNA as a novel biomarker of efficacy for dose-finding designs in oncology Supplemental material, sj-pdf-1-smm-10.1177_09622802251350457 for Using circulating tumor DNA as a novel biomarker of efficacy for dose-finding designs in oncology by Xijin Chen, Pavel Mozgunov, Richard D Baird and Thomas Jaki in Statistical Methods in Medical Research

sj-png-2-smm-10.1177_09622802251350457 - Supplemental material for Using circulating tumor DNA as a novel biomarker of efficacy for dose-finding designs in oncology Supplemental material, sj-png-2-smm-10.1177_09622802251350457 for Using circulating tumor DNA as a novel biomarker of efficacy for dose-finding designs in oncology by Xijin Chen, Pavel Mozgunov, Richard D Baird and Thomas Jaki in Statistical Methods in Medical Research

sj-png-3-smm-10.1177_09622802251350457 - Supplemental material for Using circulating tumor DNA as a novel biomarker of efficacy for dose-finding designs in oncology Supplemental material, sj-png-3-smm-10.1177_09622802251350457 for Using circulating tumor DNA as a novel biomarker of efficacy for dose-finding designs in oncology by Xijin Chen, Pavel Mozgunov, Richard D Baird and Thomas Jaki in Statistical Methods in Medical Research

sj-png-4-smm-10.1177_09622802251350457 - Supplemental material for Using circulating tumor DNA as a novel biomarker of efficacy for dose-finding designs in oncology Supplemental material, sj-png-4-smm-10.1177_09622802251350457 for Using circulating tumor DNA as a novel biomarker of efficacy for dose-finding designs in oncology by Xijin Chen, Pavel Mozgunov, Richard D Baird and Thomas Jaki in Statistical Methods in Medical Research

sj-png-5-smm-10.1177_09622802251350457 - Supplemental material for Using circulating tumor DNA as a novel biomarker of efficacy for dose-finding designs in oncology Supplemental material, sj-png-5-smm-10.1177_09622802251350457 for Using circulating tumor DNA as a novel biomarker of efficacy for dose-finding designs in oncology by Xijin Chen, Pavel Mozgunov, Richard D Baird and Thomas Jaki in Statistical Methods in Medical Research
