# Supplementary material for: Using circulating tumor DNA as a novel biomarker of efficacy for dose-finding designs in oncology
Source: Stat Methods Med Res. 2025 Jul 1;34(8):1665–83. doi: 10.1177/09622802251350457 (PMC12365363; doi:10.1177/09622802251350457)
Supplement: sj-pdf-1-smm-10.1177_09622802251350457 - Supplemental material for Using circulating tumor DNA as a novel biomarker of efficacy for dose-finding designs in oncology [file sj-pdf-1-smm-10.1177_09622802251350457.pdf]

Appendices

A Marginal distribution given only efficacy

For the joint distribution $\pi_{a,b}(\pi_T(d_j),\ \pi_E(d_j))$, the corresponding marginal distribution after marginalization based on parameters related to efficacy outcomes E is $\phi(\pi_T(d_j),\ a) = (\pi_T(d_j))^a(1-\pi_T(d_j))^{(1-a)}$. For the following formula for joint distribution, we want to integrate out $\pi_E(d_j)$ and ψ and $b = 0$ (no efficacy outcomes) holds. We use π_T and π_E as shorthand for $\pi_T(d_j)$ and $\pi_E(d_j)$ in the following context.

$$\pi_{a,b}(\pi_T,\ \pi_E) = (\pi_T)^a(1-\pi_T)^{(1-a)}(\pi_E)^b(1-\pi_E)^{(1-b)}+ \tag{11}$$

$$(-1)^{(a+b)}(\pi_E)(1-\pi_E)(\pi_T)(1-\pi_T)\frac{e^\psi - 1}{e^\psi + 1}. \tag{12}$$

$$\int\int \pi_{a,b}(\pi_T,\ \pi_E)d\pi_E d\psi \tag{13}$$

$$= \int \left[(\pi_T)^a(1-\pi_T)^{(1-a)}(\pi_E)^b(1-\pi_E)^{(1-b)}\right]\ d\pi_E + \tag{14}$$

$$\int\int \left[(-1)^{(a+b)}(\pi_E)(1-\pi_E)(\pi_T)(1-\pi_T)(\frac{e^\psi - 1}{e^\psi + 1})\right]\ d\pi_E d\psi. \tag{15}$$

$$\int_0^1 \left[(\pi_T)^a(1-\pi_T)^{(1-a)}(\pi_E)^b(1-\pi_E)^{(1-b)}\right]\ d\pi_E \tag{16}$$

$$= B(b+1)(2-b)(\pi_T)^a(1-\pi_T)^{(1-a)} \tag{17}$$

$$= \frac{1}{2}(\pi_T)^a(1-\pi_T)^{(1-a)} \tag{18}$$

$$\int_{-\infty}^{\infty}\int_0^1 \left[(-1)^{(a+b)}(\pi_E)(1-\pi_E)(\pi_T)(1-\pi_T)(\frac{e^\psi - 1}{e^\psi + 1})\right]\ d\pi_E d\psi \tag{19}$$

$$= (-1)^{(a+b)}B(2,2)(\pi_T)(1-\pi_T)\int_{-\infty}^{\infty}(\frac{e^\psi - 1}{e^\psi + 1})d\psi \tag{20}$$

$$\int_{-\infty}^{\infty}(\frac{e^\psi - 1}{e^\psi + 1})d\psi \tag{21}$$

$$= \int_{-\infty}^{\infty}\frac{e^\psi - 1 + e^\psi - e^\psi}{e^\psi + 1}d\psi \tag{22}$$

$$= \int_{-\infty}^{\infty}\frac{2e^\psi - (e^\psi + 1)}{e^\psi + 1}d\psi \tag{23}$$

$$= (2\ln(e^\psi + 1) - \psi + C)\Big|_{-\infty}^{\infty} \tag{24}$$

Since function $f(\psi) = 2\ln(e^\psi + 1) - \psi$ is an even function, which mean that $f(x) = f(-x)$. Therefore, $(2\ln(e^\psi + 1) - \psi + C)\Big|_{-\infty}^{\infty} = 0$ and $\int_{-\infty}^{\infty}(\frac{e^\psi - 1}{e^\psi + 1})d\psi = 0$

Therefore, it is safe to use the marginal distribution $(\pi_T)^a(1-\pi_T)^{(1-a)}$ as the corresponding likelihood when there are no evaluable efficacy outcomes, considering the constant does not matter.

B Utility of investigated doses under all investigated scenarios

	T_0E_0	T_0E_1	T_0E_2	T_0E_3	T_0E_4		T_0E_5	T_0E_6	T_0E_7	T_0E_8	T_0E_9
d_1	-0.712	-0.712	-0.712	-0.712	-0.302	d_1	-0.784	-0.712	-0.712	-0.712	-0.302
d_2	-0.677	-0.608	-0.608	-0.131	-0.290	d_2	-0.775	-0.608	-0.608	-0.131	-0.290
d_3	-0.648	-0.583	-0.109	-0.109	-0.287	d_3	-0.648	-0.519	-0.398	-0.287	-0.287
d_4	-0.674	-0.185	-0.185	-0.185	-0.311	d_4	-0.865	-0.736	-0.614	-0.501	-0.614
	T_1E_0	T_1E_1	T_1E_2	T_1E_3	T_1E_4		T_1E_5	T_1E_6	T_1E_7	T_1E_8	T_1E_9
d_1	-0.537	-0.537	-0.537	-0.537	**-0.056**	d_1	-0.618	-0.537	-0.537	-0.537	**-0.056**
d_2	-0.517	-0.44	-0.44	0.132	-0.069	d_2	-0.626	-0.440	-0.440	0.132	-0.069
d_3	-0.499	-0.426	0.137	0.137	-0.085	d_3	-0.499	-0.355	-0.216	-0.085	-0.085
d_4	-0.500	0.086	0.086	0.086	-0.074	d_4	-0.712	-0.569	-0.432	-0.302	-0.432
	T_2E_0	T_2E_1	T_2E_2	T_2E_3	T_2E_4		T_2E_5	T_2E_6	T_2E_7	T_2E_8	T_2E_9
d_1	-0.506	-0.506	-0.506	-0.506	-0.009	d_1	-0.589	-0.506	-0.506	-0.506	-0.009
d_2	-0.441	-0.360	-0.360	**0.280**	**0.045**	d_2	-0.556	-0.360	-0.360	**0.280**	**0.045**
d_3	-0.468	-0.394	0.195	0.195	-0.041	d_3	-0.468	-0.320	-0.177	-0.041	-0.041
d_4	-0.462	0.153	0.153	0.153	-0.019	d_4	-0.679	-0.533	-0.392	-0.258	-0.392
	T_3E_0	T_3E_1	T_3E_2	T_3E_3	T_3E_4		T_3E_5	T_3E_6	T_3E_7	T_3E_8	T_3E_9
d_1	-0.506	-0.506	-0.506	-0.506	-0.009	d_1	-0.589	-0.506	-0.506	-0.506	-0.009
d_2	-0.430	-0.348	-0.348	0.303	0.062	d_2	-0.546	-0.348	-0.348	**0.303**	0.062
d_3	-0.360	-0.278	**0.429**	**0.429**	**0.124**	d_3	-0.360	**-0.197**	**-0.035**	0.124	**0.124**
d_4	-0.426	0.218	0.218	0.218	0.034	d_4	-0.648	-0.499	-0.355	-0.216	-0.355
	T_4E_0	T_4E_1	T_4E_2	T_4E_3	T_4E_4		T_4E_5	T_4E_6	T_4E_7	T_4E_8	T_4E_9
d_1	-0.506	-0.506	-0.506	-0.506	-0.009	d_1	-0.589	-0.506	-0.506	-0.506	-0.009
d_2	-0.430	-0.348	-0.348	0.303	0.062	d_2	-0.546	-0.348	-0.348	**0.303**	0.062
d_3	-0.360	-0.278	0.429	0.429	0.124	d_3	-0.360	**-0.197**	**0.035**	0.124	**0.124**
d_4	-0.294	**0.517**	**0.517**	**0.517**	**0.250**	d_4	-0.537	-0.375	0.214	-0.056	-0.214

Table 4: Utility of different doses with $c = 2.07$, $\pi^*_{1,E} = 0.4$ and $\pi^*_{2,T} = 0.7$. The utility of the OBD are denoted in bold.

C Best dose and good dose levels for the evaluation of the WagesTait and BMI-WagesTait approaches

	E_0	E_1	E_2	E_3	E_4
T_0	–	–	–	–	–
T_1	–	–	–	–	d_1
T_2	–	–	–	d_2	d_2
T_3	–	–	d_3	d_3	d_3
T_4	–	d_4	d_4	d_4	d_4

	E_5	E_6	E_7	E_8	E_9
T_0	–	–	–	–	–
T_1	–	–	–	–	d_4
T_2	–	–	–	d_2	d_2
T_3	–	d_3	d_3	d_2	d_3
T_4	–	d_3	d_3	d_2	d_3

Table 5: Best dose of all scenarios. The scenarios supposed to end up with early stopping are notated with a hyphen.

	E_0	E_1	E_2	E_3	E_4
T_0	–	–	–	–	–
T_1	–	–	–	–	d_1
T_2	–	–	–	d_2	$(d_1 - d_2)$
T_3	–	–	d_3	$(d_2 - d_3)$	$(d_1 - d_3)$
T_4	–	d_4	$(d_3 - d_4)$	$(d_2 - d_4)$	$(d_1 - d_4)$

	E_5	E_6	E_7	E_8	E_9
T_0	–	–	–	–	–
T_1	–	–	–	–	d_4
T_2	–	–	–	d_2	$(d_1 - d_2)$
T_3	–	$(d_2 - d_3)$	d_3	$(d_2 - d_3)$	$(d_1 - d_3)$
T_4	–	d_3	$(d_3 - d_4)$	$(d_2 - d_4)$	$(d_1 - d_4)$

Table 6: Good dose(s) of all scenarios. The scenarios supposed to end up with early stopping are notated with a hyphen.

	E_0	E_1	E_2	E_3	E_4
T_0	0.000 (–)	0.001 (–)	0.001 (–)	0.000 (–)	-0.008 (–)
T_1	0.016 (–)	0.017 (–)	0.002 (–)	0.003 (–)	-0.002 (**d1**)
T_2	0.000 (–)	0.034 (–)	0.023 (–)	-0.001 (**d2**)	-0.001 (**d1-d2**)
T_3	-0.034 (–)	-0.006 (–)	0.044 (**d3**)	0.034 (**d2-d3**)	-0.010 (**d1-d3**)
T_4	0.050 (–)	0.022 (**d4**)	-0.016 (**d3-d4**)	0.003 (**d2-d4**)	-0.003 (**d1-d4**)

Table 7: Average difference of the BMI-WagesTait from the WagesTait in the proportion of correct decision (**Good dose**) under all monotonicity scenarios with a fixed $\sigma = 5$ and $\tau = 0.5$. For scenarios without a good dose, the proportion of early stopping is illustrated, while for scenarios with good dose(s), PCS is demonstrated.

	E_5	E_6	E_7	E_8	E_9
T_0	0.000 (–)	0.001 (–)	0.000 (–)	-0.001 (–)	-0.008 (–)
T_1	0.005 (–)	0.032 (–)	0.021 (–)	0.023 (–)	0.000 (**d4**)
T_2	0.040 (–)	0.020 (–)	-0.009 (–)	0.018 (**d2**)	0.001 (**d1-d2**)
T_3	0.002 (–)	0.045 (**d2-d3**)	0.012 (**d3**)	0.034 (**d2-d3**)	-0.005 (**d1-d3**)
T_4	0.013 (–)	0.012 (**d3**)	0.003 (**d3-d4**)	-0.006 (**d2-d4**)	0.002 (**d1-d4**)

Table 8: Average difference of the BMI-WagesTait from the WagesTait in the proportion of correct decision (**Good dose**) under all umbrella scenarios with a fixed $\sigma = 5$ and $\tau = 0.5$. For scenarios without a good dose, the proportion of early stopping is illustrated, while for scenarios with good dose(s), PCS is demonstrated.

D Proportion of recommendation of the EffTox and BMI-EffTox approaches

	T_0E_0		T_0E_1		T_0E_2		T_0E_3		T_0E_4	
	EffTox	*BMI-EffTox*	*EffTox*	*BMI-EffTox*	*EffTox*	*BMI-EffTox*	*EffTox*	*BMI-EffTox*	*EffTox*	*BMI-EffTox*
d_1	0	0	0.003	0.002	0.001	0.001	0.019	0.012	0.035	0.033
d_2	0	0	0.002	0	0.002	0.003	0.005	0.002	0.004	0.005
d_3	0	0	0	0	0	0.001	0.002	0.001	0.004	0.003
d_4	0	0	0	0	0	0	0	0	0.004	0.004
	T_1E_0		T_1E_1		T_1E_2		T_1E_3		T_1E_4	
	EffTox	*BMI-EffTox*	*EffTox*	*BMI-EffTox*	*EffTox*	*BMI-EffTox*	*EffTox*	*BMI-EffTox*	*EffTox*	*BMI-EffTox*
d_1	0.023	0.011	0.055	0.028	0.036	0.020	0.153	0.110	**0.464**	**0.453**
d_2	0.036	0.019	0.217	0.160	0.402	0.400	0.506	0.504	0.387	0.380
d_3	0.039	0.042	0.115	0.123	0.125	0.126	0.096	0.117	0.073	0.072
d_4	0.017	0.013	0.022	0.015	0.016	0.013	0.013	0.012	0.021	0.020
	T_2E_0		T_2E_1		T_2E_2		T_2E_3		T_2E_4	
	EffTox	*BMI-EffTox*	*EffTox*	*BMI-EffTox*	*EffTox*	*BMI-EffTox*	*EffTox*	*BMI-EffTox*	*EffTox*	*BMI-EffTox*
d_1	0.027	0.014	0.046	0.030	0.007	0.002	0.002	0.002	0.166	0.196
d_2	0.044	0.029	0.269	0.200	0.484	0.460	**0.657**	**0.629**	**0.607**	**0.585**
d_3	0.171	0.111	0.335	0.330	0.403	0.424	0.319	0.341	0.210	0.195
d_4	0.042	0.036	0.040	0.042	0.031	0.025	0.015	0.017	0.013	0.014
	T_3E_0		T_3E_1		T_3E_2		T_3E_3		T_3E_4	
	EffTox	*BMI-EffTox*	*EffTox*	*BMI-EffTox*	*EffTox*	*BMI-EffTox*	*EffTox*	*BMI-EffTox*	*EffTox*	*BMI-EffTox*
d_1	0.040	0.027	0.021	0.013	0.002	0	0	0	0.152	0.158
d_2	0.016	0.020	0.038	0.028	0.023	0.017	0.087	0.063	0.200	0.182
d_3	0.292	0.223	0.676	0.683	**0.819**	**0.833**	**0.814**	**0.833**	**0.544**	**0.553**
d_4	0.190	0.169	0.240	0.222	0.152	0.141	0.098	0.104	0.104	0.105
	T_4E_0		T_4E_1		T_4E_2		T_4E_3		T_4E_4	
	EffTox	*BMI-EffTox*	*EffTox*	*BMI-EffTox*	*EffTox*	*BMI-EffTox*	*EffTox*	*BMI-EffTox*	*EffTox*	*BMI-EffTox*
d_1	0.031	0.024	0.014	0.018	0.004	0.002	0	0	0.137	0.172
d_2	0.015	0.014	0.033	0.027	0.020	0.012	0.087	0.055	0.178	0.170
d_3	0.082	0.068	0.148	0.115	0.314	0.292	0.486	0.426	0.310	0.279
d_4	0.715	0.649	**0.793**	**0.821**	**0.657**	**0.688**	**0.425**	**0.518**	**0.374**	**0.378**

Table 9: Average proportion of recommendations per dose in both EffTox and BMI-EffTox approaches with monotonic efficacy scenarios with a fixed $\sigma = 5$ and $\tau = 0.5$. The results of OBD are denoted in bold.

	$T0E5$		$T0E6$		$T0E7$		$T0E8$		$T0E9$	
	EffTox	*BMI-EffTox*	*EffTox*	*BMI-EffTox*	*EffTox*	*BMI-EffTox*	*EffTox*	*BMI-EffTox*	*EffTox*	*BMI-EffTox*
d_1	0.001	0	0.007	0.004	0.008	0.005	0.027	0.020	0.036	0.034
d_2	0.000	0	0.001	0.001	0.002	0.002	0.005	0.005	0.006	0.008
d_3	0.000	0	0.001	0.001	0.001	0.000	0.003	0.002	0.005	0.005
d_4	0.000	0	0.001	0.001	0.001	0.000	0.002	0.001	0.004	0.004
	$T1E5$		$T1E6$		$T1E7$		$T1E8$		$T1E9$	
	EffTox	*BMI-EffTox*	*EffTox*	*BMI-EffTox*	*EffTox*	*BMI-EffTox*	*EffTox*	*BMI-EffTox*	*EffTox*	*BMI-EffTox*
d_1	0.038	0.015	0.164	0.086	0.095	0.055	0.238	0.176	**0.558**	**0.510**
d_2	0.032	0.021	0.166	0.142	0.296	0.249	0.434	0.456	0.344	0.353
d_3	0.024	0.018	0.043	0.050	0.096	0.103	0.083	0.091	0.048	0.064
d_4	0.017	0.010	0.023	0.018	0.021	0.017	0.021	0.015	0.012	0.012
	$T2E5$		$T2E6$		$T2E7$		$T2E8$		$T2E9$	
	EffTox	*BMI-EffTox*	*EffTox*	*BMI-EffTox*	*EffTox*	*BMI-EffTox*	*EffTox*	*BMI-EffTox*	*EffTox*	*BMI-EffTox*
d_1	0.055	0.020	0.124	0.078	0.039	0.021	0.070	0.037	0.242	0.221
d_2	0.052	0.042	0.285	0.243	0.394	0.321	**0.634**	**0.667**	**0.591**	**0.597**
d_3	0.117	0.088	0.175	0.159	0.335	0.320	0.246	0.218	0.154	0.156
d_4	0.023	0.015	0.025	0.019	0.028	0.031	0.024	0.020	0.007	0.007
	$T3E5$		$T3E6$		$T3E7$		$T3E8$		$T3E9$	
	EffTox	*BMI-EffTox*	*EffTox*	*BMI-EffTox*	*EffTox*	*BMI-EffTox*	*EffTox*	*BMI-EffTox*	*EffTox*	*BMI-EffTox*
d_1	0.094	0.040	0.179	0.101	0.034	0.021	0.058	0.032	0.287	0.246
d_2	0.054	0.055	0.122	0.138	0.063	0.068	**0.147**	**0.202**	0.236	0.261
d_3	0.198	0.162	**0.354**	**0.343**	**0.677**	**0.661**	0.649	0.613	**0.435**	**0.460**
d_4	0.113	0.074	0.097	0.080	0.164	0.141	0.136	0.127	0.039	0.024
	$T4E5$		$T4E6$		$T4E7$		$T4E8$		$T4E9$	
	EffTox	*BMI-EffTox*	*EffTox*	*BMI-EffTox*	*EffTox*	*BMI-EffTox*	*EffTox*	*BMI-EffTox*	*EffTox*	*BMI-EffTox*
d_1	0.146	0.056	0.262	0.134	0.043	0.018	0.048	0.032	0.346	0.258
d_2	0.062	0.074	0.119	0.154	0.046	0.050	**0.128**	**0.159**	0.222	0.272
d_3	0.125	0.131	**0.218**	**0.254**	**0.248**	**0.258**	0.311	0.286	**0.304**	**0.353**
d_4	0.246	0.171	0.216	0.192	0.644	0.630	0.510	0.515	0.125	0.112

Table 10: Average proportion of recommendations per dose in both EffTox and BMI-EffTox approaches under umbrella-shaped efficacy scenarios with a fixed $\sigma = 5$ and $\tau = 0.5$. The results of OBD are denoted in bold.

E Differences between the EffTox and BMI-EffTox approaches in the proportion of correct decision

	E_0	E_1	E_2	E_3	E_4
T_0	0.000 (–)	0.002 (–)	0.000 (–)	0.009 (–)	0.000 (–)
T_1	0.070 (–)	0.072 (–)	0.024 (–)	0.019 (–)	-0.011 (**d1**)
T_2	0.090 (–)	0.098 (–)	0.017 (–)	-0.028 (**d2**)	-0.022 (**d2**)
T_3	0.002 (–)	0.002 (–)	0.014 (**d3**)	0.019 (**d3**)	0.009 (**d3**)
T_4	0.081 (–)	0.028 (**d4**)	0.031 (**d4**)	0.093 (**d4**)	0.004 (**d4**)

Table 11: Average difference of the BMI-EffTox from the EffTox in the proportion of correct decision under with monotonic efficacy scenarios with a fixed $\sigma = 5$ and $\tau = 0.5$. For scenarios without an OBD, the proportion of early stopping is illustrated, while for scenarios with OBD, PCS is demonstrated.

	E_5	E_6	E_7	E_8	E_9
T_0	-0.001 (–)	-0.001 (–)	-0.001 (–)	0.005 (–)	-0.002 (–)
T_1	0.033 (–)	0.080 (–)	0.061 (–)	0.045 (–)	-0.048 (**d1**)
T_2	0.050 (–)	0.104 (–)	0.057 (–)	0.033 (**d2**)	0.006 (**d2**)
T_3	0.002 (–)	-0.011 (**d3**)	-0.016 (**d3**)	0.055 (**d2**)	0.025 (**d3**)
T_4	0.135 (–)	0.036 (**d3**)	0.010 (**d3**)	0.031 (**d2**)	0.049 (**d3**)

Table 12: Average difference of the BMI-EffTox from the EffTox in the proportion of correct decision under umbrella efficacy scenarios with fixed $\sigma = 5$ and $\tau = 0.5$. For scenarios without an OBD, the proportion of early stopping is illustrated, while for scenarios with OBD, PCS is demonstrated.

F The duration of treatment of the EffTox and BMI-EffTox approaches

	T_0E_0		T_0E_1		T_0E_2		T_0E_3		T_0E_4	
	EffTox	*BMI-EffTox*	*EffTox*	*BMI-EffTox*	*EffTox*	*BMI-EffTox*	*EffTox*	*BMI-EffTox*	*EffTox*	*BMI-EffTox*
d_1	12	7.150	12	7.175	12	7.225	12	7.175	12	9.350
d_2	12	7.750	12	8.100	12	8.100	12	10.300	12	9.550
d_3	12	8.075	12	8.425	12	10.675	12	10.775	12	9.900
d_4	12	8.250	12	11.125	12	11.175	12	11.100	12	10.100

	T_1E_0		T_1E_1		T_1E_2		T_1E_3		T_1E_4	
	EffTox	*BMI-EffTox*	*EffTox*	*BMI-EffTox*	*EffTox*	*BMI-EffTox*	*EffTox*	*BMI-EffTox*	*EffTox*	*BMI-EffTox*
d_1	12	7.250	12	7.150	12	7.200	12	7.200	12	9.300
d_2	12	7.675	12	8.075	12	8.075	12	10.275	12	9.575
d_3	12	8.075	12	8.400	12	10.725	12	10.750	12	9.800
d_4	12	8.450	12	11.225	12	11.175	12	11.150	12	10.325

	T_2E_0		T_2E_1		T_2E_2		T_2E_3		T_2E_4	
	EffTox	*BMI-EffTox*	*EffTox*	*BMI-EffTox*	*EffTox*	*BMI-EffTox*	*EffTox*	*BMI-EffTox*	*EffTox*	*BMI-EffTox*
d_1	12	7.200	12	7.125	12	7.200	12	7.150	12	9.275
d_2	12	7.675	12	8.075	12	8.025	12	10.275	12	9.525
d_3	12	8.100	12	8.375	12	10.775	12	10.750	12	9.800
d_4	12	8.425	12	11.175	12	11.250	12	11.175	12	10.150

	T_3E_0		T_3E_1		T_3E_2		T_3E_3		T_3E_4	
	EffTox	*BMI-EffTox*	*EffTox*	*BMI-EffTox*	*EffTox*	*BMI-EffTox*	*EffTox*	*BMI-EffTox*	*EffTox*	*BMI-EffTox*
d_1	12	7.200	12	7.125	12	7.150	12	7.200	12	9.275
d_2	12	7.650	12	8.025	12	8.025	12	10.250	12	9.525
d_3	12	8.075	12	8.400	12	10.775	12	10.775	12	9.800
d_4	12	8.425	12	11.125	12	11.225	12	11.200	12	10.325

	T_4E_0		T_4E_1		T_4E_2		T_4E_3		T_4E_4	
	EffTox	*BMI-EffTox*	*EffTox*	*BMI-EffTox*	*EffTox*	*BMI-EffTox*	*EffTox*	*BMI-EffTox*	*EffTox*	*BMI-EffTox*
d_1	12	7.175	12	7.175	12	7.175	12	7.200	12	9.30
d_2	12	7.625	12	8.025	12	8.075	12	10.275	12	9.55
d_3	12	8.000	12	8.400	12	10.775	12	10.775	12	9.75
d_4	12	8.375	12	11.175	12	11.200	12	11.200	12	10.30

Table 13: Average duration of treatment in weeks for BMI-EffTox and EffTox across monotonic efficacy scenarios with fixed parameters $\sigma = 5$ and $\tau = 0.5$.

	T_0E_5		T_0E_6		T_0E_7		T_0E_8		T_0E_9	
	EffTox	*BMI-EffTox*	*EffTox*	*BMI-EffTox*	*EffTox*	*BMI-EffTox*	*EffTox*	*BMI-EffTox*	*EffTox*	*BMI-EffTox*
**d1**	12	6.532	12	7.208	12	7.311	12	7.154	12	9.272
**d2**	12	6.968	12	8.002	12	8.124	12	10.376	12	9.556
**d3**	12	8.058	12	8.376	12	9.277	12	8.561	12	9.829
**d4**	12	6.938	12	6.920	12	8.863	12	9.186	12	8.124
	T_1E_5		T_1E_6		T_1E_7		T_1E_8		T_1E_9	
	EffTox	*BMI-EffTox*	*EffTox*	*BMI-EffTox*	*EffTox*	*BMI-EffTox*	*EffTox*	*BMI-EffTox*	*EffTox*	*BMI-EffTox*
**d1**	12	6.556	12	7.196	12	7.106	12	7.085	12	9.318
**d2**	12	6.965	12	8.075	12	8.003	12	10.310	12	9.569
**d3**	12	8.205	12	8.444	12	9.350	12	8.754	12	9.882
**d4**	12	7.173	12	7.155	12	8.708	12	9.152	12	7.984
	T_2E_5		T_2E_6		T_2E_7		T_2E_8		T_2E_9	
	EffTox	*BMI-EffTox*	*EffTox*	*BMI-EffTox*	*EffTox*	*BMI-EffTox*	*EffTox*	*BMI-EffTox*	*EffTox*	*BMI-EffTox*
**d1**	12	6.473	12	7.080	12	7.211	12	7.082	12	9.297
**d2**	12	6.910	12	8.097	12	8.071	12	10.283	12	9.619
**d3**	12	8.106	12	8.362	12	9.332	12	8.702	12	9.827
**d4**	12	7.256	12	7.145	12	8.792	12	9.285	12	7.963
	T_3E_5		T_3E_6		T_3E_7		T_3E_8		T_3E_9	
	EffTox	*BMI-EffTox*	*EffTox*	*BMI-EffTox*	*EffTox*	*BMI-EffTox*	*EffTox*	*BMI-EffTox*	*EffTox*	*BMI-EffTox*
**d1**	12	6.554	12	7.111	12	7.165	12	7.270	12	9.256
**d2**	12	7.016	12	8.077	12	8.079	12	10.244	12	9.526
**d3**	12	8.077	12	8.436	12	9.269	12	8.678	12	9.817
**d4**	12	7.222	12	7.247	12	8.765	12	9.222	12	8.051
	T_4E_5		T_4E_6		T_4E_7		T_4E_8		T_4E_9	
	EffTox	*BMI-EffTox*	*EffTox*	*BMI-EffTox*	*EffTox*	*BMI-EffTox*	*EffTox*	*BMI-EffTox*	*EffTox*	*BMI-EffTox*
**d1**	12	6.457	12	7.143	12	7.178	12	7.108	12	9.375
**d2**	12	6.911	12	7.974	12	8.079	12	10.295	12	9.518
**d3**	12	8.004	12	8.395	12	9.360	12	8.757	12	9.816
**d4**	12	7.242	12	7.157	12	8.616	12	9.374	12	8.005

Table 14: Average duration of treatment in weeks for BMI-EffTox and EffTox across umbrella-shaped efficacy scenarios with fixed parameters $\sigma = 5$ and $\tau = 0.5$.

G Differences between approaches in the proportion of toxicity

	E_0	E_1	E_2	E_3	E_4
T_0	0.006	-0.009	-0.025	0.015	-0.009
T_1	-0.007	0.004	0.005	0.005	-0.002
T_2	-0.003	0.010	0.010	0.012	-0.002
T_3	-0.005	0.006	0.008	0.011	0.001
T_4	0.000	0.002	0.003	0.006	-0.001

	E_5	E_6	E_7	E_8	E_9
T_0	0.003	0.004	-0.001	0.001	-0.002
T_1	0.015	0.017	0.018	0.017	0.007
T_2	0.011	0.010	0.011	0.009	0.006
T_3	0.004	0.001	0.006	0.004	-0.003
T_4	0.002	0.004	0.004	0.001	0.000

Table 15: Average difference of the BMI-EffTox from the EffTox in the proportion of toxicity under all scenarios with a fixed $\sigma = 5$ and $\tau = 0.5$.

	E_0	E_1	E_2	E_3	E_4
T_0	0.001	0.003	0.005	0.004	0.0097
T_1	-0.003	0.001	0.002	0.001	-0.002
T_2	0.001	0.001	-0.001	-0.002	-0.003
T_3	0.001	0.004	-0.001	0.002	-0.002
T_4	-0.004	0.003	-0.002	-0.003	-0.001

	E_5	E_6	E_7	E_8	E_9
T_0	0.001	0.003	0.003	0.003	0.007
T_1	0.002	-0.001	0.001	0.002	-0.005
T_2	-0.003	-0.002	0.000	0.001	-0.001
T_3	0.001	0.001	0.000	0.002	-0.001
T_4	-0.002	-0.001	0.001	-0.003	0.001

Table 16: Average difference of the BMI-WagesTait from the WagesTait in the proportion of toxicity under all scenarios with a fixed $\sigma = 5$ and $\tau = 0.5$.

H Between-subject variance of ctDNA values

Trajectories of $\mu_{t,j}^{(i)}$ and corresponding changes of $\pi_{\mathrm{Cmax}\leq 10}$ with time of different doses are investigated in Figure 2 in Section 3.2. We investigate the behaviour of ctDNA under different simulations settings and different values of σ in the distribution $C_{t,j}^{(i)} \sim \mathcal{N}(\mu_{t,j}^{(i)},\ \sigma^2),\ t \in [0, 12]$ in Figure 8, where the within-subject correlation is fixed at $\tau = 0$, assuming that there is no within-patient variance.

The impact of between-subject variance of ctDNA values σ varies according to the reaction to treatment, such as, 'response to treatment' or 'treatment resistance'. The amount of information or the contribution by ctDNA can be quantified by the value of $\pi_{\mathrm{Cmax}\leq\epsilon}$ as discussed in Section 3.2. In Figure 8, ctDNA is expected to provide all of the information about efficacy before week 4 in the case of response to treatment (setting 2) with $\sigma = 1$ ($\pi_{\mathrm{Cmax}\leq\epsilon} = \pi_E(d_j)$ before week 4). However, that is not the same case under the setting of treatment resistance (setting 1) with $\sigma = 1$, where $\pi_{\mathrm{Cmax}\leq\epsilon} = 1$ before week 8. A larger value $\sigma = 10$ will lead to more information than a small σ does under the setting of treatment resistance (setting 1), but less information in the case of response to treatment (setting 2). This indicates that the impact of σ on the trajectories of $\mu_{t,j}^{(i)}$ and $\pi_{\mathrm{Cmax}\leq\epsilon}$ depends on different settings. In other words, the amount of information or the contribution of ctDNA depends on both the level of noise and specific contexts.

Figure 8: Trajectory of $\mu_{t,j}^{(i)}$ and $\pi_{\mathrm{Cmax}\leq 10}$ for a specific dose with $\pi_E(d_j) = 0.3$ under different settings and two different values of σ. The value $\tau = 0$ and $s^{(i)}$ are fixed to obtain fixed $\mu_{t,j}$ for a particular patient i.

I Intra-subject correlation of ctDNA values

For the impact of intra-subject correlation $s_j^{(i)}$ in Equation (7), ctDNA plays different roles under different values of intra-patient variance. More information based on ctDNA is expected under smaller values of τ.

For illustration, the proportion of recommendations per dose under $\tau = 5$ is investigated as shown in Figure 9. In the presence of a significant intra-patient variation (i.e., $\tau = 5$), de-escalation of dose recommendation is more likely to happen via the BMI-EffTox, which indicates that high noise in ctDNA will make the BMI-EffTox more conservative than the original EffTox. This is more likely to happen under scenarios with fewer constraints, such as scenarios with T_4. The noise brought by a significant intra-patient variation is less likely to bring benefits in terms of the PCS in contrast to the results shown in Figure 3 under $\tau = 0.5$.

Figure 9: Average differences of the BMI-EffTox from the EffTox in the proportion of recommendation under monotonic efficacy scenarios with a fixed $\sigma = 5$ and $\tau = 5$. Scenarios with no OBD are denoted with a grey background.

Unexpectedly, there are more increases in the proportion of recommendations, namely, decreases in the proportion of early stopping under most of the investigated scenarios without an OBD in Figure 9. For this reason, we explored the impact of τ on the proportion of early stopping. We choose scenarios under T_1 with more strict safety constraints, which are representative as an example to demonstrate the impact of τ on the results of the proportion of early stopping. In Figure 10, ctDNA values generated with small τ (i.e., $\tau = 0$) will encourage early stopping rather than preventing early stopping as in the case of large τ (i.e., $\tau = 5$). When compared with the results of EffTox approach indicated by the red line, the contribution of ctDNA on the proportion of early stopping is the opposite. Specifically, if the information from ctDNA is concentrated enough, early stopping will be encouraged. Otherwise, the BMI-EffTox encourages to spend more time on exploring different doses.

Figure 10: Average proportion of early stopping via the BMI-EffTox under T_1 with a fixed $\sigma = 5$ and different values of τ. The red line indicates the results of the proportion of early stopping via EffTox.

In Figure 11, we observe smaller differences in treatment duration between active and inactive doses at high τ values, due to increased noise. That is, the reduction in treatment duration becomes less pronounced when intra-subject variance is large.

Overall, incorporating ctDNA in dose-finding studies effectively accounts for intra-patient variance. High intra-patient variance tends to prevent early stopping or dose escalation, in contrast to the effects observed with low intra-patient variance.

Figure 11: Distribution of the duration of treatment of the BMI-EffTox and the EffTox under monotonic efficacy scenarios with a fixed $\sigma = 5$ and different values of τ.
