# Supplementary figures and images for: Using circulating tumor DNA as a novel biomarker of efficacy for dose-finding designs in oncology
Source: Stat Methods Med Res. 2025 Jul 1;34(8):1665–83. doi: 10.1177/09622802251350457 (PMC12365363; doi:10.1177/09622802251350457)

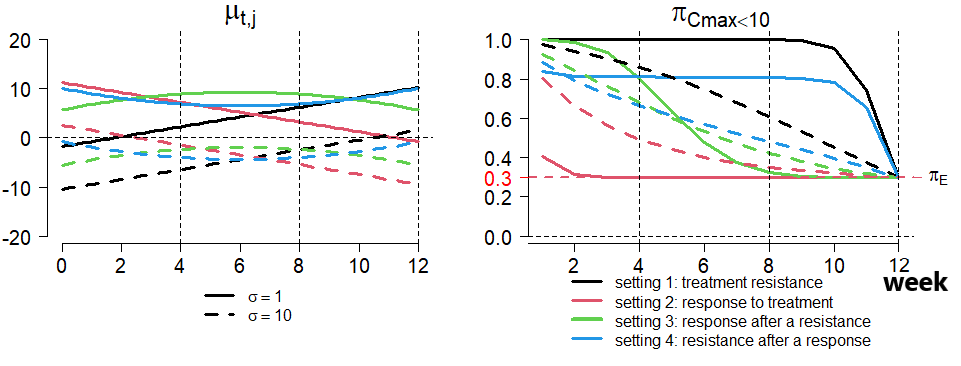

Supplement: sj-png-2-smm-10.1177_09622802251350457 - Supplemental material for Using circulating tumor DNA as a novel biomarker of efficacy for dose-finding designs in oncology [file sj-png-2-smm-10.1177_09622802251350457.png]

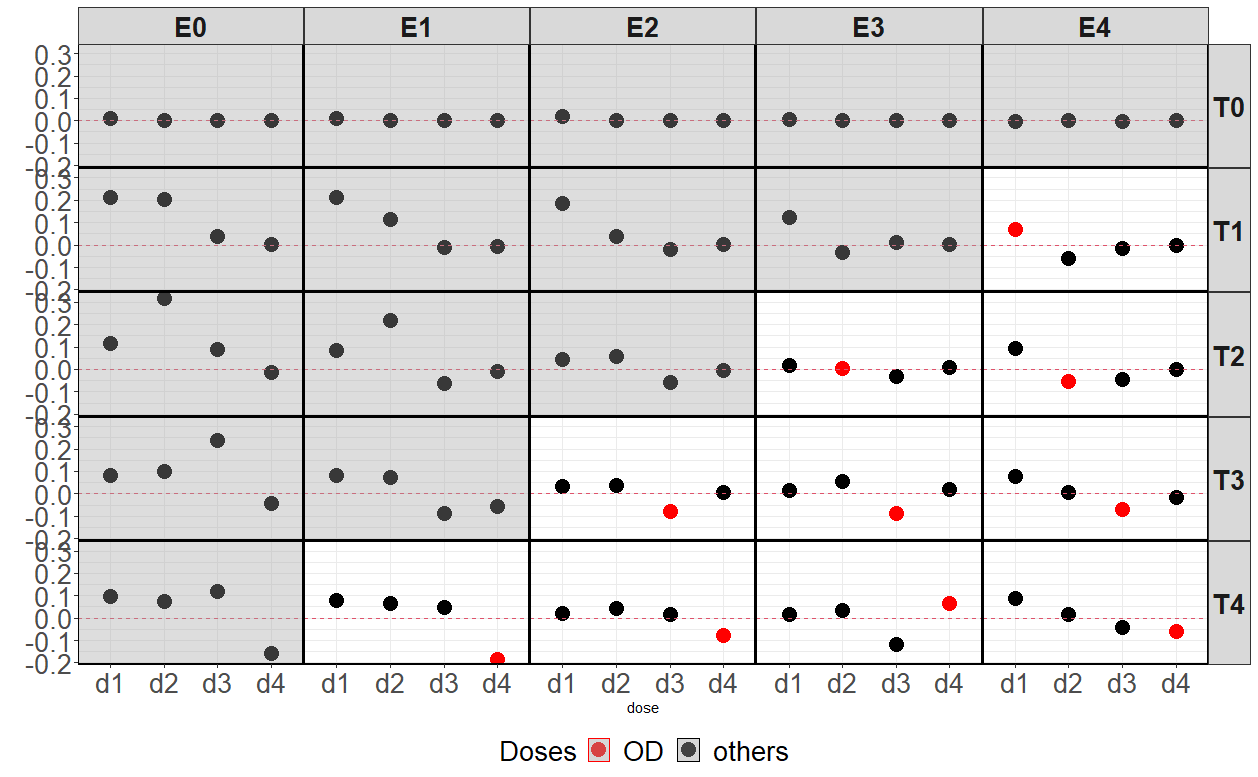

Supplement: sj-png-3-smm-10.1177_09622802251350457 - Supplemental material for Using circulating tumor DNA as a novel biomarker of efficacy for dose-finding designs in oncology [file sj-png-3-smm-10.1177_09622802251350457.png]

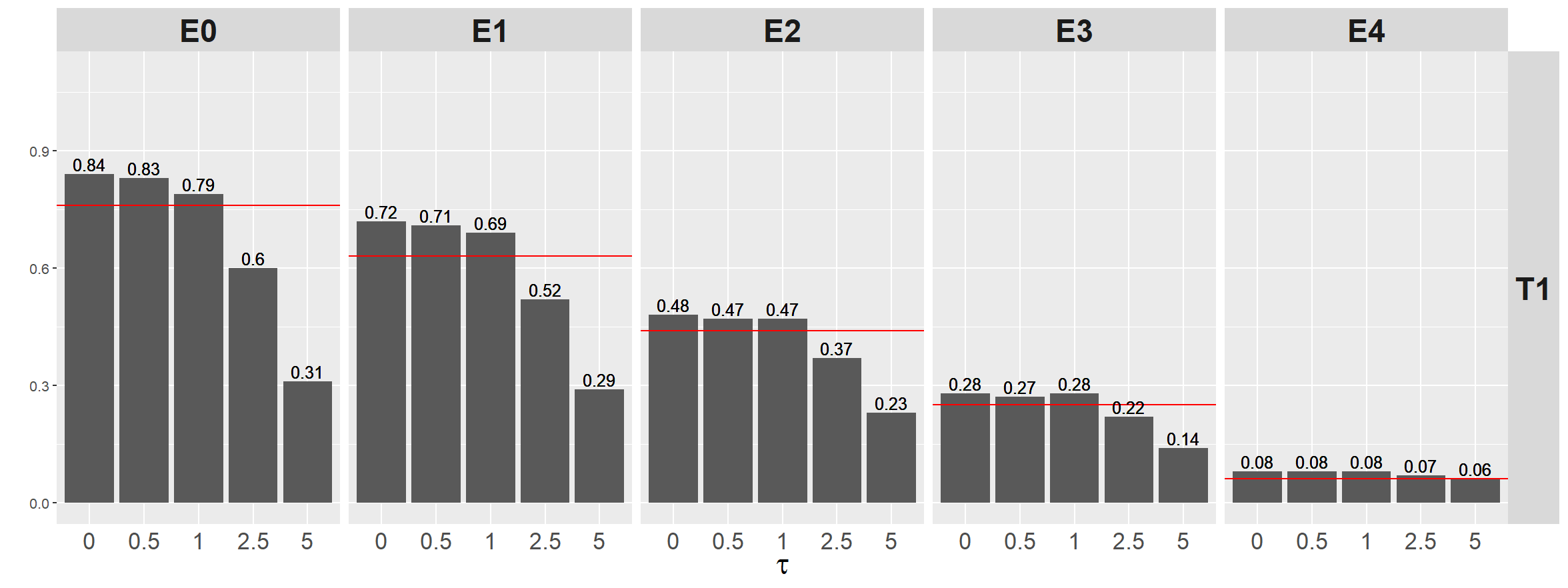

Supplement: sj-png-4-smm-10.1177_09622802251350457 - Supplemental material for Using circulating tumor DNA as a novel biomarker of efficacy for dose-finding designs in oncology [file sj-png-4-smm-10.1177_09622802251350457.png]

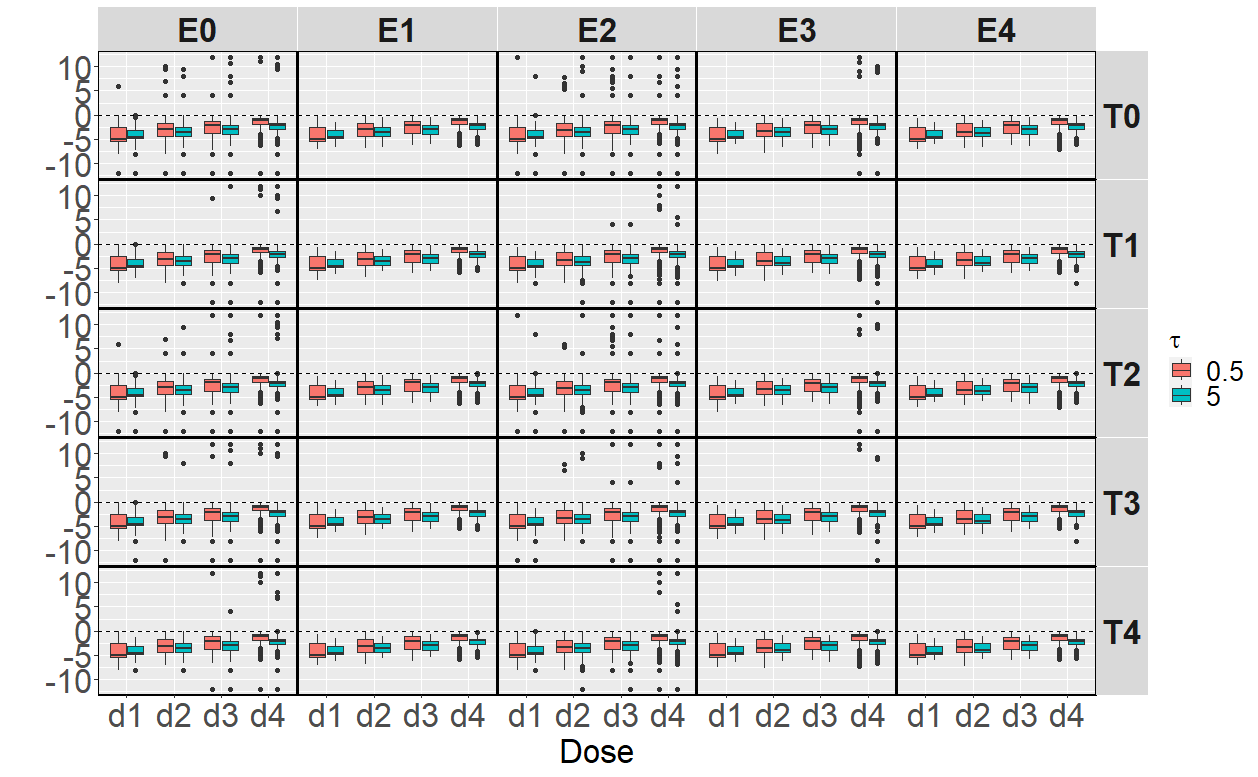

Supplement: sj-png-5-smm-10.1177_09622802251350457 - Supplemental material for Using circulating tumor DNA as a novel biomarker of efficacy for dose-finding designs in oncology [file sj-png-5-smm-10.1177_09622802251350457.png]
